# Perceptions towards COVID-19 and adoption of preventive measures among the public in Saudi Arabia: a cross sectional study

**DOI:** 10.1186/s12889-021-11223-8

**Published:** 2021-06-29

**Authors:** Ghadah Alkhaldi, Ghadeer S. Aljuraiban, Sultana Alhurishi, Roberta De Souza, Kethakie Lamahewa, Rosa Lau, Fahdah Alshaikh

**Affiliations:** 1 The Department of Community Health Sciences, College of Applied Medical Sciences, King Saud University, Riyadh, Kingdom of Saudi Arabia; 2 Research Chair of Health Education and Health Promotion, Department of Family and Community Medicine, College of Medicine, King Saud University, Riyadh, Saudi Arabia; 3 Department of Statistics, Universidade Federal de São Carlos, São Carlos, Brazil; 4 Institute for Research and Development Sri Lanka, Battaramulla, Sri Lanka; 5 Camden and Islington Public Health, Islington Council, London, UK

## Abstract

**Background:**

Effective management of the spread of a novel infectious disease, such as the COVID-19 virus can be achieved through influencing people’s behavior to adopt preventive measures. The public’s perceptions and attitudes towards the virus, governmental guidance and preventive measures were unknown in Saudi Arabia.

**Objectives:**

1) investigate the public perception of COVID-19, anxiety level, the COVID-19 information sources sought, adoption of preventive measures, and ability and willingness to self-isolate during and post-lockdown periods of the COVID-19 pandemic in Saudi Arabia; 2) investigate socio-demographic factors associated with adoption of preventive measures against COVID-19 and self-isolation practices.

**Method:**

Between April 22nd and June 21st 2020, Saudi adults aged ≥18 years voluntarily completed a self-administered web-based cross-sectional survey, distributed through social media (WhatsApp) and emails to representatives in education, health, business, and social sectors across all Saudi Arabian regions. The survey included questions on anxiety level, COVID-19 risk perceptions and adoption of preventive measures. Weighted percentages, Pearson’s chi-square tests, and multiple logistic regression were applied to evaluate associations between these factors and socio-demographic variables.

**Results:**

A total of 2393 respondents completed the survey. A majority (74%) were worried about the COVID-19 outbreak and of those, 27% reported that it was likely that they would be infected with COVID-19; 16% believed it would be life-threatening or severe. However, only 11% of respondents reported high anxiety level. Adoption of hygiene practices and social distancing were lower among older (> 65 years) compared to younger (18–24 years) respondents (OR: 0.06; 95% CI: 0.01, 0.28 and OR 0.06; 95% CI: 0.01, 0.27 respectively). High percentages of respondents reported being able to (88%) and were willing to (82%) self-isolate. Those with the lowest gross household income and those with at least one flu symptom were less able and willing to self-isolate. A significant increase in levels of anxiety, perceived effectiveness of social distancing and hygiene practices was reported in the post-lockdown compared to during the lockdown.

**Conclusions:**

The study reported high levels of adoption of preventive measures, willingness and perceived ability to self-isolate during the early phase of the pandemic. Vulnerable groups such as the elderly, and those with low socio-economic status reported lower adoption of preventive measures or ability and willingness to self-isolate. Tailored public health messages and interventions are needed to achieve high adherence to these preventive measures in these groups.

**Supplementary Information:**

The online version contains supplementary material available at 10.1186/s12889-021-11223-8.

## Background

On December 31st 2020, a pneumonia of unknown cause in Wuhan China was first reported to the World Health Organization (WHO) [1]. On January 12th, 2020, the WHO declared the cause to be a novel coronavirus called “2019-nCoV” [1]; the name was subsequently changed to “SARS-CoV-2” by the International Committee on Taxonomy of Viruses on February 11th, 2020 [2]. The WHO formally named the disease caused by this novel virus “COVID-19” [1].

As of 2nd Febraury, 2021, the current cumulative confirmed cases in Saudi Arabia number is around 368,000, with 360,000 recovered cases and a total of 6379 deaths due to COVID-19 [3, 4].

Saudi Arabia started a widespread awareness campaign in early February and followed with a set of gradual system-level suppressive measures (e.g. lockdown andcontact tracing) once the first case was announced on March 2nd 2020. Examples of such suppressive measures included closure of schools and worksites. Suppressive measures had shown short term success in China and South Korea [5]. The key aim of suppressive measures was to reduce the average number of secondary cases each COVID-19 case generated, known as the reproduction number or R, to below one. This was intended to reduce the number of cases or eliminate human-to-human transmission [5]. However, as this was a temporary measure to reduce the peak of the COVID-19 outbreak, the question remained for how long and how many times such measures would need to be enacted. Applying these suppressive measures over a long period of time was thought to be likely to have a substantial economic and social impact [6, 7].

Research into individuals’ risk perception is essential to understanding their response, behavior, and adoption of individual-level preventive measures (e.g. wearing masks, washing hands) in case of an infectious disease outbreak and its aftermath. Identifying risk perception will not only help mitigate the devastating mortality and morbidity burden, but also economic loss. With the relatively long period needed for the majority of the population to be vaccinated against COVID-19 and the emergence of new COVID-19 variants [8–11], understanding and addressing behavior to reduce transmission and spread of infection is imperative [12, 13] to avoid further spikes of new cases and unavoidable enforcement of lockdowns [14]. Such insight will help identify population groups with relatively low risk perceptions or low adoption of preventive measures and enable the design of policies and interventions tailored to these populations. It will allow governments to strengthen key public health messages and design health awareness campaigns tailored to the different stages of an outbreak [5, 15–18]. Furthermore, gaining insights into risk perception and behaviors can help build community resilience and influence behavior to increase uptake of future vaccination and/or treatment [5, 13, 15–18].

In Saudi Arabia, there is limited research on how people behave or perceive the risk of previous infectious disease outbreaks [19]. Our study addressed this important question in relation to the current COVID-19 pandemic. By understanding the Saudi community’s perceived vulnerability and fear of COVID-19 as well as their behavior, it will be possible to develop tailored interventions to encourage adoption of preventive measures and manage anxiety and fear. Hence, this study aimed to identify the anxiety level, risk perception, information sources, adoption of preventive measures, and self-isolation practices during and post-lockdown in a sample of Saudi adults. Additionally, this study aimed to explore factors associated with adoption of preventive measures against COVID-19 and self-isolation practices.

## Methods

### Study design

Between April 22nd and June 21st 2020, adults aged 18 years and older from the general public who were willing to participate and had been living in Saudi Arabia for at least a week, were invited to complete an open web-based survey during the COVID-19 pandemic in Saudi Arabia. The survey was hosted on SURVS with full General Data Protection Regulation (GDPR) coverage [20]. Multiple entries from the same individual were prevented through authentication cookies. Each section was displayed separately, and respondents had to respond to each item before moving on to the next section. Respondents could go back to edit previous answers, but once they submitted the survey they were not allowed to change any of their responses. On average, respondents spent 15 min to complete the survey.

Convenience sampling was used to reach a large number of Saudi adults online, two online platforms were used including WhatsApp and E-mails. Invitation messages to complete the survey that included brief information about the study and its link were first distributed by the local researchers through lists of selected WhatsApp contacts and groups, with a request to recirculate it. Additionally, a request to circulate the survey to their mailing lists was sent to different institutions and organizations representing the main sectors in the community: education (e.g. public and private universities), health (e.g. Saudi Arabia Center for Disease Control), business (e.g. Council of Saudi Chambers), and social (e.g. Family Affairs Council) sectors across all Saudi Arabian regions. A web-based survey was used as it was likely to capture a greater number of harder-to-reach individuals than a paper survey [21, 22] especially during the lockdown period where access to people was limited . In addition, according to the Saudi Communication and Information Technology Commission, WhatsApp was the most widely used social media application [23]. A 2017 report by Statista showed that Saudi Arabia’s WhatsApp penetration rate was around 73%, which is one of the highest worldwide [24].

### Sample size calculation and weighting

For a representative sample of the Saudi Arabian adult population, the proportions of gender and age groups in the population (obtained from the General Authority for Statistics [25]) were considered for the sample size estimate. The smallest population proportion is female over 75 years old (0.71%). Size n was estimated by normal statistical test for a proportion with significance level α = 5%. As a result, a sample size of 2180 is sufficient to cover all gender and age ranges of the adult population. Age and gender were considered for post-stratification weights to compensate for the fact that people with certain characteristics were not as likely to respond to the survey [26]. Non-response weights were also used to compensate for bias in the final sample. The weight that combines age and gender is calculated by the rate between population and initial sample proportions of each age and gender range (%). The initial sample corresponds to 4067 individuals that answered the survey (complete and incomplete responses). The weight of the non-response is the rate of the proportion in the initial sample to the proportion in the final sample (complete responses). The final weight is the product of both weights. The weighted final sample size was 2393 corresponding to individuals who completed the survey.

### Instrument description

The survey was originally designed for the COVID-19 outbreak in Hong Kong (HK) by public health experts from the Chinese University of HK [17] and translated into English and used by experts at Imperial College London, United kingdom (UK) [27, 28]. The survey was translated and adapted to the Saudi Arabian context using the WHO instrument translation process [29] and validated by a panel of academic researchers. Agreement scores on the translation were obtained by three bilingual researchers (GA, FA and SA) with experience in survey development and methodology. All the researchers used a Likert scale of 5 points with the values 4 and 5 corresponding to substantial or strong agreement, respectively. The Kappa value, which was significantly different from zero indicated agreement (*p* = 0.004) and the agreement scores presented a mean = 89.6%, median = 91.7%, min = 75% and max = 100% implying an almost perfect agreement. Next, pretesting and cognitive interviews were conducted on a subgroup of 23 respondents (not included in final analysis) following the WHO steps for translating instruments and to ensure all questions and responses adapted to the Saudi Arabian context were clear [29].

An Arabic and English version were distributed. The survey consisted of four main sections and 46 items (See Additional file 1 for survey).

The survey included:

1. Socio-demographic characteristics: age group (18–24, 25–34,35-44,45-54,55-64,65–74,75 years old and above), gender (male, female), pregnancy, marital status (married, separated/divorced, widowed, single), region of residence in Saudi Arabia in the last 7 days (13 region of Saudi Arabia), educational or work-related qualification (read and write, primary, intermediate, secondary/equivalent, pre-univ.diploma, university, high diploma, master, PhD, prefer not to say), nationality (Saudi, non-Saudi, prefer not to say), employment (working fulltime (≥ 30 h/week), working part time (8–29 h a week), working part time (< 8 h a week), full-time student, retired, unemployed, not working and other), health worker, gross household income (under 1333USD per month, 1333 USD to 2666 USD per month, 2666.67 USD to 3999.74 USD per month, 4000USD to 5333.07 USD per month, 5333.34 USD to 6666.41 USD per month, 6666.67 USD and over per month, don’t know, prefer not to say), care giving responsibilities (child (ren) aged under 5, child (ren) aged 5 to 16, elderly relative/ dependent, disabled dependent, not applicable, prefer not to say, other), perceived current state of health (very good, fairly good, neither good or poor, fairly poor, very poor, prefer not to say), chronic health conditions in the last 6 months (i.e. since October 2019), chronic condition in members of the household or those under your care, and respiratory/ cold/ flu-like symptoms in the last 14 days (persistent fever, shivering, headache, muscle pain, cough, difficulty in breathing or shortness of breath, dizziness, runny nose, sore throat, not applicable, prefer not to say).
2. Anxiety levels assessed using the validated Arabic version of the Hospital Anxiety and Depression Scale (HADS) [30, 31].
3. Perceptions of different sources of Information regarding COVID-19 and where respondents accessed information related to COVID-19.
4. Perceptions, behaviors, and attitude in relation to COVID-19 and its prevention measures.
  This was assessed by asking respondents about the following:

- worry about Covid-19 and history of testing for infection.
- perceived susceptibility to and severity of being infected with COVID-19 under Saudi Arabia’s current measures (risk perceptions).
- adoption of preventive measures which included:
  - adoption of different preventive measures to protect self-and/or others. These preventive measures were categorized into three groups following the surveys used in the UK and HK [17, 27, 28]: hygiene practices, social distancing, and travel avoidance. And were analyzed through three variables which represent at least one adopted measure taken for each of these categories. These measures include wearing face masks, washing hands with soap and water regularly, using hand sanitizer more regularly and covering one’s nose and mouth when sneezing or coughing for hygiene practices; avoiding contact with people who have a fever or respiratory symptoms or who have been in affected areas in the last 14 days, avoiding going out in general, crowded areas, going into public markets that sell fresh fish, meat, and poultry products, going to hospitals or other healthcare settings, using public transport, going into shops and supermarkets, going to work and social events while social distancing; and avoiding travel to affected countries and areas inside and outside Saudi Arabia, regardless of whether they were affected for travel avoidance.
  - reasons for adoption of preventive measures.
  - perceived effectiveness of preventive measures. The responses for this part of the survey were for effective measures they corresponded to responses: very effective or fairly effective. While An ineffective measure corresponded to response fairly ineffective or very ineffective or ‘don’t know’.
- willingness and/or ability to self-isolate, which was defined in that period as not leaving home (even to buy food or essentials) or having any visitors for 14 days if the person returned from traveling abroad from affected countries or came in contact with an infected person.

Context in relation to the Covid-19 pandemic and its policies

The day before the survey dissemination (i.e. 21st April 2020), the government announced that restrictions during Ramadan-the Islamic holy month- would be relaxed, after a complete nationwide lockdown for around 20 days [32]. On April 26th 2020, movement within cities were allowed, except for Mecca, between 9 am till 5 pm with certain conditions (e.g. no social gatherings of more than 5 people etc.). Breaking of curfew would result in a substantial fine and repeated offenders would be given prison sentences. Permits were given to those who needed it and essential workers during curfew hours. People were allowed a walking hour /day during curfew, conditional to the same residential neighborhood. On May 31st 2020, lockdown was eased (free movement between 6 am-8 pm within and between regions). People were allowed to go back to work, mosques were opened, and domestic traveling was resumed. On June 21st, 2020, lockdown was completely lifted. Starting from 6 am, life went back to normal with certain guidelines, penalties and precautions [32].

### Data analysis

Descriptive analysis was carried out: number (n), percentage (%) and the weighted percentage (%w). Pearson’s chi-square test was applied for the associations between responses and time period. Univariable and multivariable logistic regression models were used to identify the associations of anxiety, risk perception, adoption of preventive measures and their perceived effectiveness, ability and willingness to self-isolate with socio-demographic factors. The univariable analysis was undertaken to help identify the potential covariates to enter in the multivariable model (*p*-value < 0.2). Odds ratios for univariable (OR^1^) and multivariable (OR) logistic models and OR 95% confidence intervals (95% C.I. OR) were estimated. For the time trend analysis, descriptive weighted relative frequencies of the responses in each time period (during lockdown and post lockdown) were used. Adjusted residuals (AR) were estimated to identify significant differences. All the analyses considered the weighted sample. The significance level of the tests α = 5%. In order to make the models fit adequately to better predict outcomes, control variables that remained in the final multivariable model to risk perception were “at least one respiratory/ cold/ flu-like symptom” and to ability to self-isolate were “gender and nationality “. Data were analyzed using the SPSS software version 22.0 (SPSS Science, Chicago, IL, USA).

## Results

### Sample description

A total of 2393 respondents completed the survey (59% completion rate), of which 19% were 18–24 years old and around 5% were 65 years old and above (Table 1). Most respondents (60%) were male, 45% university graduates, 52% worked full time, and 71% were in Riyadh (Saudi Arabia’s capital) in the last 7 days before responding to the survey. Around 83% of the respondents had some kind of care giving responsibility towards children or dependent elderly, and 79% reported that their health was very good. Around 11 and 14% of the population reported high anxiety and depression, respectively in the last 14 days before filling up the survey (See Additional file 2).

**Table 1:** Descriptive Statistics Socio-Demographic Characteristics (Section I in the survey): absolute (n), relative (%) and weighted relative (%w) frequencies

	***n***	***%***	***%w***
**Age group**			
18–24	920	38.4	19.4
25–34	636	26.6	25.7
35–44	469	19.6	27.2
45–54	227	9.5	15.9
55–64	120	5.0	7.5
65–74	19	0.8	2.8
75 years old and above	2	0.1	1.5
**Gender**			
Male	927	38.7	59.9
Female	1466	61.3	40.1
**Marital Status**			
Married	1033	43.2	60.3
Separated/ Divorced	86	3.6	4.0
Widowed	18	0.8	0.7
Single	1256	52.5	35.0
**Region of Residence in Saudi Arabia in the last 7 days**			
Makkah	349	14.6	13.0
Riyadh	1654	69.1	70.8
Asir	23	1.0	0.8
Jawf	2	0.1	0.0
Northern Borders	3	0.1	0.2
Bahah	9	0.4	0.2
Madinah	20	0.8	0.8
Ha’il	9	0.4	0.5
Najran	1	0.0	0.1
Qasim	17	0.7	0.8
Tabuk	20	0.8	1.1
Jizan	37	1.5	2.0
Eastern Province	237	9.9	8.5
Outside Saudi Arabia	8	0.3	0.4
Other	4	0.2	0.8
**Educational or Work- related Qualification**			
Read and Write	1	0.0	0.0
Primary	1	0.0	0.1
Intermediate	4	0.2	0.2
Secondary/Equivalent	258	10.8	7.1
Pre-Univ.Diploma	90	3.8	5.6
University	1272	53.2	45.1
High Diploma	27	1.1	1.7
Master	447	18.7	20.7
PhD	286	12.0	19.2
Prefer not to say	7	0.3	0.3
**Nationality**			
Saudi	2160	90.3	85.8
Non-Saudi	211	8.8	12.6
Prefer not to say	22	0.9	1.6
**Employment**			
Working fulltime (≥ 30 h/week)	919	38.4	52.1
Working part time (8–29 h a week)	100	4.2	5.4
Working part time (< 8 h a week)	64	2.7	2.9
Fulltime student	819	34.2	21.1
Retired	82	3.4	6.1
Unemployed	65	2.7	2.0
Not working	259	10.8	6.8
Other	85	3.6	3.6
**Gross Household Income**			
Under 1333 USD per month	149	6.2	4.9
1333 USD to 2666 USD per month	254	10.6	10.4
2667.67 USD to 3999.74 USD per month	363	15.2	17.1
4000 USD to 5333.07 USD per month	281	11.7	12.9
5333.34 USD to 6666.41 USD per month	200	8.4	8.9
6666.67 USD and over per month	549	22.9	25.5
Don’t know	342	14.3	8.5
Prefer not to say	255	10.7	11.8
**Care Giving Responsibilities**			
Child (ren) aged under 5	532	22.2	27.6
Child (ren) aged 5 to 16	756	31.6	38.8
Elderly relative/ dependent	358	15.0	16.4
Disabled dependent	52	2.2	2.3
Not applicable	1015	42.4	32.7
Prefer not to say	123	5.1	5.0
Other	106	4.4	6.7
**Perceived Current State of Health**			
Very good	1832	76.6	78.6
Fairly good	404	16.9	15.4
Neither good or poor	83	3.5	2.7
Fairly poor	42	1.8	1.3
Very poor	10	0.4	0.4
Prefer not to say	22	0.9	1.6
**Chronic Health Conditions in the Last 6 Months (i.e. Since October 2019)**			
Eye conditions	41	1.7	1.5
Ear, nose and/ or throat condition	222	9.3	8.6
Cancer	7	0.3	0.4
Epilepsy/ seizure	8	0.3	0.3
Stroke	1	0.0	0.0
Hypertension	120	5.0	7.2
Heart disease	13	0.5	0.7
Asthma	131	5.5	4.6
Emphysema, bronchitis, bronchiectasis	10	0.4	0.4
Tuberculosis	2	0.1	0.0
Thyroid glands disease	107	4.5	4.5
Diabetes mellitus	100	4.2	7.6
Hyperlipidaemia	41	1.7	2.1
Kidney condition	17	0.7	0.7
Liver condition	6	0.3	0.3
Bowel condition	59	2.5	2.7
Anaemia	227	9.5	5.8
Genetic blood disorders	9	0.4	0.2
Skeletomuscular disorders	55	2.3	2.5
Autoimmune disorder	19	0.8	0.8
Skin condition	111	4.6	3.9
Depression	193	8.1	6.5
Anxiety	297	12.4	9.6
Schizophrenia	7	0.3	0.9
Not applicable	1291	53.9	53.6
Prefer not to say	51	2.1	2.4
Other	34	1.4	1.4
**Chronic Condition in Members of the Household or Those Under Your Care**			
Yes, they do	1109	46.3	39.8
No, they don’t	1146	47.9	54.8
Don’t know	112	4.7	3.7
Prefer not to say	26	1.1	1.7
**Respiratory/ Cold/ Flu-like Symptoms in the Last 14 Days**			
Persistent fever	23	1.0	0.8
Shivering	15	0.6	0.4
Headache	370	15.5	11.5
Muscle pain	115	4.8	4.3
Cough	94	3.9	3.3
Difficulty in breathing or shortness of breath	78	3.3	2.3
Dizziness	107	4.5	2.7
Runny nose	156	6.5	5.3
Sore throat	135	5.6	6.0
Not applicable	1770	74.0	77.7
Prefer not to say	28	1.2	1.1
	**2393**	**100.0**	**100.0**

Most respondents (94%) were not tested for COVID-19 (See Additional file 2). Information about COVID-19 was mostly obtained through official websites (72%) such as local governmental agencies and the WHO and their social media outlets; 70% of respondents perceived this information source as very reliable while 48% viewed unofficial websites as very unreliable. The second most popular source of information was social media platform (48%). Around 57% of respondents would like to receive the latest research explaining what is known about coronavirus from a trusted source. At least 78% believe that coronavirus is most likely transmitted through physical contact with someone who has the virus with or without symptoms. Around 31% believed that it was very unlikely that transmission could happen through consumption of meat made of wild animals.

### Risk perception and anxiety

Overall most of the respondents were worried about the COVID-19 outbreak in Saudi Arabia (75%) (See Additional file 2, Table 2). Of those, 27% reported that it was likely that they would be infected with COVID-19; 16% believed it would be life-threatening or severe and 38% expected it to be moderate. Older respondents (aged 35 years and older) were less likely to worry about COVID-19 and their perceived susceptibility and severity were lower compared to the younger respondents (aged between 18 and 24 years) (Table 2). The worry about COVID-19 was less in those with the highest gross household income of 6666.67 USD and over,compared to those in the lowest gross household income of under 1333 USD. The perceived susceptibility and severity was higher in those with the highest gross household income compared to those with the lowest. Respondents aged between 45 and 64 years old and those with gross household income of 4000 USD per month and over were less anxious compared to younger respondents and those in the lowest gross household income groups, respectively (See Table 3).

**Table 2:** Risk perception odds ratio estimates (OR^1^ and OR) and OR 95% confidence interval (95% C.I. OR) of the multiple logistic regression model

	Risk Perception											
	Worried about COVID19				Perceived suspebitblity (likely to be infected)				Perceived severity (expect at least a moderate infection)			
Socio-demographic factors	OR^**1**^	OR	95% C.I.OR	***p***-value	OR^**1**^	OR	95% C.I.OR	***p***-value	OR^**1**^	OR	95% C.I.OR	***p***-value
**Age Group**												
**18–24**												
25–34	1.04	0.878	(0.628–1.226)	0.445	1.32**	0.866	(0.641–1.168)	0.345	0.90	0.796	(0.603–1.049)	0.105
35–44	0.84*	**0.621**	**(0.415–0.929)**	**0.020**	1.24*	**0.638**	**(0.447–0.912)**	**0.014**	0.7**	**0.600**	**(0.45–0.8)**	**< 0.001**
45–54	0.47**	**0.366**	**(0.234–0.571)**	**< 0.001**	1.17	**0.623**	**(0.417–0.931)**	**0.021**	0.69**	**0.659**	**(0.473–0.92)**	**0.014**
55–64	0.58**	**0.436**	**(0.258–0.737)**	**0.002**	0.87	**0.438**	**(0.271–0.707)**	**0.001**	0.61**	**0.537**	**(0.351–0.822)**	**0.004**
**65 years old and above**	0.34**	**0.313**	**(0.17–0.574)**	**< 0.001**	0.65*	**0.410**	**(0.224–0.75)**	**0.004**	0.82	1.275	(0.733–2.215)	0.390
**Gender**												
**Female**	1.54**	**1.704**	**(1.375–2.111)**	**< 0.001**	0.97				0.96			
**Marital status**												
**Single**												
**Married**	0.92	**1.586**	**(1.155–2.179)**	**0.004**	1.18*	**1.522**	**(1.157–2)**	**0.003**	0.87*			
Separated/ Divorced	0.67*	0.938	(0.551–1.6)	0.816	1.5*	**1.988**	**(1.22–3.24)**	**0.006**	0.76*			
Widowed	0.70	0.846	(0.273–2.619)	0.771	1.06	1.706	(0.577–5.044)	0.334	0.65			
**Educational or Work- related Qualification**												
**PhD**												
Until Secondary/Equivalent	1.99**				0.57**	**0.524**	**(0.349–0.788)**	**0.002**	1.28*	1.217	(0.811–1.825)	0.342
Pre-Univ.Diploma	1.56**				0.36**	**0.340**	**(0.218–0.531)**	**< 0.001**	1.22	1.413	(0.932–2.141)	0.104
University	1.68**				0.68**	**0.600**	**(0.458–0.785)**	**< 0.001**	1.49**	**1.503**	**(1.154–1.957)**	**0.003**
High Diploma	1.74*				0.89	0.807	(0.41–1.587)	0.534	0.62*	0.686	(0.338–1.392)	0.296
Master	1.7**				1.01	0.852	(0.638–1.137)	0.276	1.68**	**1.765**	**(1.326–2.35)**	**< 0.001**
**Nationality**												
**Saudi**	1.77**	**1.433**	**(1.076–1.909)**	**0.014**	1.01				1.22*			
**Employment**												
**Unemployed/Not working/Other**												
**Working/Student full time**	1.25*	**1.512**	**(1.11–2.059)**	**0.009**	1.74**	**1.348**	**(1.029–1.767)**	**0.030**	1.67**	**1.756**	**(1.348–2.287)**	**< 0.001**
Working part time	1.36*	**1.630**	**(1.037–2.561)**	**0.034**	1.13	0.981	(0.66–1.458)	0.926	1.01	1.077	(0.73–1.591)	0.708
Retired	0.97	**1.772**	**(1.055–2.976)**	**0.031**	0.97	1.039	(0.643–1.679)	0.876	1.06	0.985	(0.616–1.577)	0.951
**Healthcare worker**	1.2*				1.73**	**1.659**	**(1.304–2.111)**	**< 0.001**	1.11			
**Gross Household Income**												
**Under 1333 USD per month**												
1333 USD to 2666 USD per month	0.82	0.836	(0.468–1.496)	0.547	1.21	1.231	(0.772–1.96)	0.382	1.09	1.160	(0.732–1.837)	0.527
2666.67 USD to 3999.74 USD per month	0.84	0.885	(0.507–1.544)	0.666	1.43*	1.485	(0.956–2.307)	0.078	1.07	1.167	(0.757–1.799)	0.483
4000 USD to 5333.07 USD per month	0.68*	0.721	(0.408–1.275)	0.261	1.6**	1.440	(0.912–2.272)	0.117	0.90	1.061	(0.677–1.663)	0.795
5333.34 USD to 6666.41 USD per month	0.46**	**0.462**	**(0.257–0.831)**	**0.010**	2.05**	**1.926**	**(1.19–3.116)**	**0.008**	1.01	1.159	(0.72–1.865)	0.543
6666.67 USD and over per month	0.55**	**0.582**	**(0.339–0.999)**	**0.050**	1.84**	**1.681**	**(1.094–2.582)**	**0.018**	1.53**	**1.838**	**(1.203–2.808)**	**0.005**
Don’t know/ Prefer not to say	0.58**	0.639	(0.373–1.095)	0.103	1.16	1.190	(0.774–1.83)	0.429	0.81	0.833	(0.546–1.272)	0.398
**Perceived Current State of Health**												
**Fairly or Very poor/ Prefer not to say**												
**Very good**	2.29**	1.639	(0.961–2.796)	0.070	1.29				1.53*	**1.674**	**(1.015–2.761)**	**0.044**
**Fairly good**	1.94**	1.321	(0.748–2.334)	0.338	1.53*				2.42**	**2.407**	**(1.416–4.092)**	**0.001**
**Neither good or poor**	6.5**	**4.098**	**(1.566–10.723)**	**0.004**	1.88*				2.76**	**2.803**	**(1.371–5.732)**	**0.005**
**At Least One Chronic Health Conditions Currently**	1.05				1.11*				1.45**	**1.445**	**(1.207–1.729)**	**< 0.001**
**Chronic Condition in Members of the Household or Those Under Your Care**	1.29**				1.25**	**1.268**	**(1.06–1.517)**	**0.010**	1.25**			
**At Least One Respiratory/ Cold/ Flu-like Symptom**	1.18*	1.247	(0.973–1.597)	0.081	1.38**	**1.439**	**(1.171–1.769)**	**0.001**	1.36**	**1.213**	**(0.984–1.496)**	**0.070**

*p-value <  0.20; ** *p*-value <  0.05. The first categories are the references. OR^1^: odds ratio estimate by univariable logistic model. *OR* odds ratio estimate by multivariable logistic model

**Table 3:** Anxiety and Depression odds ratio estimates (OR^1^ and OR) and OR 95% confidence interval (95% C.I. OR) of the multiple logistic regression model

	Anxiety				Depression			
Socio-demographic factors	OR^**1**^	OR	95% C.I.OR	***p***-value	OR^**1**^	OR	95% C.I.OR	***p***-value
**Age Group**								
**18–24**								
**25–34**	0.97	1.146	(0.828–1.587)	0.411	1.25*	**1.357**	**(1.005–1.833)**	**0.047**
**35–44**	0.65**	0.792	(0.559–1.122)	0.190	1.00	1.060	(0.776–1.449)	0.714
45–54	0.32**	**0.483**	**(0.308–0.757)**	**0.002**	0.88	0.952	(0.661–1.37)	0.790
**55–64**	0.14**	**0.229**	(0.108–0.484)	**< 0.001**	0.44**	**0.445**	**(0.272–0.727)**	**0.001**
**65 years old and above**	0.57**	0.751	(0.365–1.546)	0.437	0.48**	**0.213**	**(0.106–0.426)**	**< 0.001**
**Gender**								
**Female**	1.49**	**1.426**	**(1.137–1.787)**	**0.002**	1.00			
**Marital status**								
**Single**								
Married	0.51**				0.71**			
Separated/ Divorced	0.65*				0.64*			
Widowed	1.11				0.97			
**Educational or Work-related Qualification**								
**PhD**								
Until Secondary/Equivalent	4.05**	**3.311**	**(1.944–5.64)**	**< 0.001**	1.37*	1.232	(0.776–1.956)	0.376
**Pre-Univ.Diploma**	4.56**	**5.290**	**(3.086–9.066)**	**< 0.001**	1.94**	**2.556**	**(1.609–4.058)**	**< 0.001**
**University**	2.5**	**2.057**	**(1.378–3.069)**	**< 0.001**	1.39**	1.311	(0.963–1.786)	0.086
High Diploma	1.71*	2.119	(0.798–5.626)	0.132	0.72	0.667	(0.283–1.574)	0.356
Master	2.67**	**2.413**	**(1.585–3.674)**	**< 0.001**	1.51**	**1.386**	**(1.004–1.914)**	**0.047**
**Nationality**								
**Saudi**	1.27*				0.69**	**0.594**	**(0.441–0.799)**	**0.001**
**Employment**								
**Unemployed/Not working/Other**								
Working/student full time	1.23*	**1.850**	**(1.301–2.632)**	**0.001**	0.94			
Working part time	0.97	1.422	(0.85–2.377)	0.180	0.69*			
**Retired**	0.19**	0.598	(0.247–1.447)	0.254	0.35**			
**Healthcare worker**	1.74**	**1.725**	**(1.302–2.285)**	**< 0.001**	1.35**	1.268	(0.99–1.624)	0.060
**Gross Household Income**								
**Under 1333 USD per month**								
**1333 USD to 2666 USD per month**	0.57**	0.622	(0.368–1.05)	0.075	1.08	1.328	(0.804–2.194)	0.268
**2666.67 USD to 3999.74 USD per month**	0.53**	0.626	(0.383–1.025)	0.063	1.15	**1.733**	**(1.072–2.802)**	**0.025**
4000 USD to 5333.07 USD per month	0.32**	**0.437**	**(0.256–0.748)**	**0.003**	0.81	1.314	(0.794–2.177)	0.288
**5333.34 USD to 6666.41 USD per month**	0.37**	**0.485**	**(0.275–0.858)**	**0.013**	0.96	1.663	(0.974–2.84)	0.063
**6666.67 USD and over per month**	0.34**	**0.545**	**(0.335–0.885)**	**0.014**	0.64**	1.128	(0.696–1.83)	0.624
Don’t know/ Prefer not to say	0.5**	**0.524**	**(0.324–0.845)**	**0.008**	0.85	1.074	(0.67–1.721)	0.767
**Perceived Current State of Health**								
**Fairly or Very poor/ Prefer not to say**								
**Very good**	0.22**	0.259	(0.151–0.443)	**< 0.001**	0.1**	**0.090**	**(0.049–0.166)**	**< 0.001**
**Fairly good**	0.65*	0.664	(0.377–1.17)	0.157	0.24**	**0.186**	**(0.099–0.35)**	**< 0.001**
Neither good or poor	1.07	1.015	(0.481–2.141)	0.968	0.54*	**0.445**	**(0.199–0.994)**	**0.048**
**At Least One Respiratory/ Cold/ Flu-like Symptom**	3.1**	2.338	(1.845–2.962)	**< 0.001**	2.35**	**1.923**	**(1.547–2.391)**	**< 0.001**

*p-value <  0.20; ** *p*-value <  0.05. The first categories are the references. . OR^1^: odds ratio estimate by univariable logistic model. *OR* odds ratio estimate by multivariable logistic model

Other socio-demographic factors were associated with higher risk perception and anxiety; being female increased the odds of worry about COVID-19 spread and anxiety by 70 and 43% compared to male, respectively and being married increased the odds of worry by 59% and the perceived susceptibility by 52%.

### Adoption of preventive measures

COVID-19 preventive measures listed in the survey (See Additional file 1 – Question 38) were adopted by nearly all of the respondents to protect themselves (98.5%). Around 98% adopted hygiene practices, 98% practiced social distancing, and 89% adopted measures related to travel avoidance. The most applied measures were washing hands more frequently with soap and water (96%), avoiding crowded areas (92%), and avoiding social events (90%) (See Table 2 in Additional file 2). Protecting others mostly involved covering one’s nose and mouth while sneezing or coughing (66%), wearing a face mask (56%), avoiding social events (52%), and washing hands more frequently with soap and water (51%).

### Hygiene practices

Being 65 years or older decreased the odds of adopting hygiene practices compared to being 18–24 years old (OR:0.06; 95% CI:0.01,0.27) (Table 4). Being married compared to single (OR:13.21; 95% CI:3.86,45.21), being Saudi compared to being of other nationality (OR: 3.15; 95% CI: 1.26,7.89), working or studying full time in relation to not working/others (OR:3.18; 95% CI:1.24,8.17), and reporting good current states of health (very good OR: 9.64; 95% CI:3.12,29.74 and fairly good OR:4.66; 95% CI:1.29,16.77) increased the odds of hygiene practices.

**Table 4:** Adoption of preventive measures odds ratio estimates (OR^1^ and OR) and OR 95% confidence interval (95% C.I. OR) for the multiple logistic regression model

	Adoption of Preventive Behaviour											
	Hygiene Practices				Social Distancing				Travel Avoidance			
**Socio-demographic factors**	**OR**^**1**^	**OR**	**95% C.I.OR**	***p*-value**	**OR**^**1**^	**OR**	**95% C.I.OR**	***p*-value**	**OR**^**1**^	**OR**	**95% C.I.OR**	***p*-value**
**Age Group**												
**18–24**												
25–34	5**	2.670	(0.684–10.427)	0.158	1.84*	0.977	(0.351–2.72)	0.964	1.21			
35–44	2.3*	0.489	(0.144–1.66)	0.251	2.22*	**0.324**	**(0.097–1.085)**	**0.068**	1.34*			
45–54	2.17*	0.314	(0.07–1.404)	0.130	1.51	**0.155**	**(0.036–0.672)**	**0.013**	1.33*			
55–64	2.62	0.298	(0.038–2.31)	0.247	1.32	**0.151**	**(0.025–0.894)**	**0.037**	1.83**			
**65 years old and above**	0.1**	**0.063**	**(0.014–0.277)**	**< 0.001**	0.09**	**0.061**	**(0.014–0.271)**	**< 0.001**	0.46**			
**Gender**												
**Female**	0.34**				0.61*	**2.433**	**(1.095–5.407)**	**0.029**	0.87			
**Marital status**												
**Single**												
**Married**	7.51**	**13.216**	**(3.863–45.212)**	**< 0.001**	6.2**	**23.410**	**(7.182–76.306)**	**< 0.001**	1.86**	**1.658**	**(1.241–2.215)**	**0.001**
Separated/ Divorced	1.10	1.972	(0.435–8.944)	0.379	1.68	3.307	(0.675–16.196)	0.140	0.96	0.872	(0.479–1.588)	0.655
Widowed	0.37*	0.592	(0.08–4.37)	0.607	0.42	0.966	(0.135–6.932)	0.972	1.08	1.043	(0.243–4.466)	0.955
**Educational or Work- related Qualification**												
**PhD**												
Until Secondary/Equivalent	2.56*				2.26*				0.72*			
Pre-Univ.Diploma	–				3*				1.62*			
University	3.45**				3.77**				1.44**			
High Diploma	–				–				2.89*			
Master	16.13**				6.35**				1.14			
**Nationality**												
**Saudi**	7.16**	**3.156**	**(1.261–7.899)**	**0.014**	5.67**	**2.882**	**(1.201–6.915)**	**0.018**	1.83**	**1.802**	**(1.273–2.553)**	**0.001**
**Employment**												
**Unemployed/Not working/Other**												
**Working/Student full time**	10.96**	**3.188**	**(1.243–8.177)**	**0.016**	7.19**	**2.597**	**(1.026–6.576)**	**0.044**	1.83**			
Working part time	2.42**	0.372	(0.108–1.287)	0.118	3.31**	0.750	(0.186–3.017)	0.686	1.55*			
Retired	12.56**	4.494	(0.426–47.424)	0.211	3.47**	0.692	(0.136–3.531)	0.658	1.26			
**Healthcare worker**	1.72				0.96	0.443	(0.181–1.083)	0.074	0.92			
**Gross Household Income**												
**Under 1333 USD per month**												
1333 USD to 2666 USD per month	2.77*				1.86				0.95			
2666.67 USD to 3999.74 USD per month	4.95**				3.12*				1.17			
4000 USD to 5333.07 USD per month	2.26*				1.06				1.00			
5333.34 USD to 6666.41 USD per month	10**				2.45				1.50			
6666.67 USD and over per month	10.9**				2.69*				0.78			
Don’t know/ Prefer not to say	0.67				0.52				0.68			
**Perceived Current State of Health**												
**Fairly or Very poor/ Prefer not to say**												
**Very good**	36.8**	**9.643**	**(3.127–29.744)**	**< 0.001**	33.49**	**10.647**	**(4.014–28.241)**	**< 0.001**	5.13**	**3.130**	**(1.816–5.394)**	**< 0.001**
**Fairly good**	18.83**	**4.661**	**(1.295–16.776)**	**0.018**	24.96**	**11.931**	**(3.59–39.652)**	**< 0.001**	4.83**	**3.238**	**(1.744–6.012)**	**< 0.001**
**Neither good or poor**	29.1**	6.444	(0.513–80.908)	0.149	10.76**	3.896	(0.777–19.52)	0.098	5.44**	**4.013**	**(1.494–10.777)**	**0.006**
**At least one chronic health conditions currently**	0.39**				0.26**	**0.436**	**(0.203–0.938)**	**0.034**	0.93			
**Chronic Condition in Members of the Household or Those Under Your Care**	1.7*				1.53*				0.72**	**0.731**	**(0.553–0.966)**	**0.027**
**At Least One Respiratory/ Cold/ Flu-like Symptom**	0.2**				0.25**				0.66**			

*p-value < 0.20; ** *p*-value < 0.05. The first categories are the references. OR^1^: odds ratio estimate by univariable logistic model. *OR* odds ratio estimate by multivariable logistic model

### Social distancing and travel avoidance

The odds of social distancing increased when the respondent was female (OR:2.43; 95% CI:1.09,5.40), married (OR:23.41; 95% CI:7.18,76.30), Saudi national (OR:2.88; 95% CI:1.20,6.91), working or studying full time (OR:2.59; 95% CI:1.02,6.57) and had a better current state of health (very good OR: 10.64; 95% CI:4.01,28.24 and fairly good OR:11.93; 95% CI:3.59,39.65), and decreased for older age (45–54 years old OR 0.15; 95% CI:0.03,0.67, 55–64 years old OR 0.15; 95% CI:0.02,0.89 and 65 + years old group OR 0.06; 95% CI:0.01,0.27) (Table 4). Being married (OR:1.65; 95% CI:1.24,2.21) or Saudi national (OR:1.80; 95% CI:1.27,2.55) increased the odds of travel avoidance. A fair or very poor current state of health or chronic health conditions in the household or responsibilities for care of others decreased the odds of travel avoidance.

### Reasons for adoption of preventive measures

Of the 10 reasons listed in the survey for adoption a preventive measure, in response to Saudi Arabia’s government guidelines was the most frequent reason, chosen by 93% of respondents, followed by 52% in response to news coverage of the outbreak, and 46% in response to the growing number of COVID-19 cases in the country (See Additional file 2).

### Perceived effectiveness of adoption of preventive measures

The perceived effectiveness of adoption of hygiene practices, social distancing, and travel avoidance were 99, 99, and 97%, respectively. At least 80% of respondents thought that washing hands frequently with soap and water, covering their noses and mouths while sneezing or coughing, avoiding contact with people who have a fever or respiratory symptoms, or have been to affected areas within the last 14 days, refraining from crowded areas, social events, and travel to affected and other areas in the world were very effective measures in preventing the spread of COVID-19.

Being Those who self-identified as female or single increased the odds of perceiving all three categories of preventive measures: hygiene practices, social distancing and travel avoidance as effective (Table 5). Having at least one respiratory/cold, flu-like symptom increased the odds of the perceived effectiveness of travel avoidance (OR: 2.79; 95% 1.12,6.97) but decreased the odds for hygiene practice (OR: 0.35; 95%0.16,0.77).

**Table 5:** Perceived Effectiveness of Preventive Measures odds ratio estimates (OR^1^ and OR) and OR 95% confidence interval (95% C.I.OR) for the multiple logistic regression model

	Perceived Effectiveness of such Behaviours											
	Hygiene Practices				Social Distancing				Travel Avoidance			
Socio-demographic factors	OR^**1**^	OR	95% C.I.OR	***p***-value	OR^**1**^	OR	95% C.I.OR	***p-***value	OR^**1**^	OR	95% C.I.OR	***p***-value
**Age Group**												
**18–24**												
25–34	2.32*				1.63				1.27			
35–44	1.39				0.46*				0.56*			
45–54	0.39**				0.34*				0.5*			
55–64	–				–				0.80			
65 years old and above	0.54				–				–			
**Gender**												
**Female**	2.7**	**4.113**	**(1.535–11.02)**	**0.005**	2.55**	**2.669**	**(1.036–6.874)**	**0.042**	3.93**	**4.417**	**(1.997–9.774)**	**< 0.001**
**Marital status**												
**Single**												
**Married**	0.70	0.769	(0.169–3.503)	0.734	0.38**	**0.321**	**(0.108–0.953)**	**0.041**	0.5**	0.828	(0.39–1.76)	0.624
**Separated/ Divorced**	0.19**	**0.109**	**(0.02–0.597)**	**0.011**	0.29*	**0.166**	**(0.028–0.976)**	**0.047**	0.33**	**0.252**	**(0.073–0.865)**	**0.028**
**Widowed**	0.19*	0.160	(0.009–2.951)	0.218	0.06**	**0.035**	**(0.004–0.307)**	**0.003**	0.15**	**0.098**	**(0.014–0.687)**	**0.019**
**Educational or Work- related Qualification**												
**PhD**												
Until Secondary/Equivalent	1.14				0.76	0.367	(0.071–1.885)	0.230	0.70	**0.357**	**(0.111–1.155)**	**0.086**
Pre-Univ.Diploma	0.33**				0.22**	**0.168**	**(0.049–0.584)**	**0.005**	0.47*	0.446	(0.15–1.332)	0.148
University	2.08*				1.05	0.599	(0.2–1.794)	0.360	1.56	0.840	(0.355–1.986)	0.691
High Diploma	–				–	–			–	–		
Master	1.28				0.85	0.560	(0.166–1.893)	0.351	0.45**	**0.313**	**(0.146–0.669)**	**0.003**
**Nationality**												
**Saudi**	0.61				1.21				3.32**	**3.174**	**(1.741–5.783)**	**0.000**
**Employment**												
**Unemployed/Not working/Other**												
Working/student full time	0.40				0.63				0.59			
Working part time	0.17**				0.26*				0.32**			
Retired	0.29*				0.45				0.33*			
**Healthcare worker**	0.65				1.13				0.98			
**Gross Household Income**												
**Under 1333 USD per month**												
1333 USD to 2666 USD per month	0.38				2.13				0.64			
2666.67 USD to 3999.74 USD per month	3.53				11.04**				0.81			
4000 USD to 5333.07 USD per month	0.32				1.42				0.68			
5333.34 USD to 6666.41 USD per month	0.40				1.72				0.80			
6666.67 USD and over per month	0.50				2.44*				0.79			
Don’t know/ Prefer not to say	0.33				1.53				0.48			
**Perceived Current State of Health**												
**Fairly or Very poor/ Prefer not to say**												
**Very good**	1.38				7.64**	**12.394**	**(4.737–32.428)**	**< 0.001**	2.22*	**3.661**	**(1.254–10.692)**	**0.018**
**Fairly good**	2.08				55.65**	**75.234**	**(5.757–983.184)**	**0.001**	5.42**	**9.020**	**(2.169–37.517)**	**0.002**
Neither good or poor	1.08				16.96*	17.434	(0.61–498.564)	0.095	1.40	1.658	(0.329–8.349)	0.540
**At Least One Chronic Health Conditions Currently**	0.5**				0.88				1.7**			
**Chronic Condition in Members of the Household or Those Under Your Care**	1.97*				1.76*				0.89			
**At Least One Respiratory/ Cold/ Flu-like Symptom**	0.5*	**0.352**	**(0.161–0.772)**	**0.009**	1.49				3.22**	**2.795**	**(1.121–6.97)**	**0.027**

*p-value < 0.20; ** *p*-value < 0.05. The first categories are the references. . OR^1^: odds ratio estimate by univariable logistic model. *OR* odds ratio estimate by multivariable logistic model

### Self-isolation

Most respondents believe they were able (88%) and willing (82%) to self-isolate, but over 40% reported that they felt the need to stock up on food supplies and toiletries in preparation (See Additional file 2). In 3 out of 5 age groups, the odds of being able to self-isolate decreased compared to the 18–24 year old group; being 25–34 year old decreased the odds by 47%, 35–44 years old decreased the odds by 53% and 65+ year old decreased the odds by 68%. Being married or retired increased the odds of being able to self-isolate compared to being single and unemployed/not working/other (OR:1.50; 95% 1.02,2.23;and 11.60;95%2.19,61.46), respectively. Four out of the five reported gross household income compared to the lowest had higher odds of being able and willing to self-isolate; for example the odds of those who reported a gross household income of 6666.67 USD per month and over were 2.1 and 1.8 time larger. At least one respiratory/ cold/ flu-like symptom decreased the odds of self-isolation by 45 and 41% to being able and willing to self-isolate, respectively (Table 6). The main worry about self-isolation was its effect on their mental health (39%) and how difficult it would be to separate themselves from those in their household (30%) (See Additional file 2). Although most respondents would report or seek help if they experienced symptoms of COVID-19, 6% reported that they would not do so if they experienced severe or moderate symptoms and 11% if they experienced mild symptoms.

**Table 6:** Self-Isolation odds ratio estimates (OR^1^ and OR) and OR 95% confidence interval (95% C.I.OR) for the multiple logistic regression model

	Self-Isolation							
	Able				Willing			
Socio-demographic factors	OR^**1**^	OR	95% C.I.OR	***p***-value	OR^**1**^	OR	95% C.I.OR	***p***-value
**Age Group**								
**18–24**								
**25–34**	0.74*	**0.532**	**(0.353–0.801)**	**0.002**	0.96	0.841	(0.613–1.154)	0.284
**35–44**	0.67**	**0.467**	**(0.286–0.762)**	**0.002**	1.00	0.990	(0.7–1.398)	0.953
45–54	1.59*	0.967	(0.519–1.805)	0.917	1.83**	**1.816**	**(1.175–2.807)**	**0.007**
**55–64**	1.73*	0.802	(0.366–1.757)	0.581	2.87**	**2.690**	**(1.457–4.965)**	**0.002**
**65 years old and above**	0.52**	**0.324**	**(0.146–0.717)**	**0.005**	0.93	0.941	(0.521–1.7)	0.841
**Gender**								
**Female**	1.01	1.266	(0.96–1.671)	0.095	0.99			
**Marital status**								
**Single**								
Married	1.39**	**1.509**	**(1.02–2.233)**	**0.039**	1.47**			
Separated/ Divorced	1.13	1.155	(0.573–2.33)	0.686	1.18			
Widowed	0.64	0.514	(0.134–1.966)	0.331	0.92			
**Educational or Work- related Qualification**								
**PhD**								
Until Secondary/Equivalent	1.06				0.77*			
**Pre-Univ.Diploma**	0.67*				0.99			
**University**	0.83				0.88			
High Diploma	1.60				1.32			
Master	0.85				1.17			
**Nationality**								
**Saudi**	1.83**	**1.424**	**(0.985–2.06)**	**0.060**	1.46**	**1.388**	**(1.009–1.909)**	**0.044**
**Employment**								
**Unemployed/Not working/Other**								
Working/student full time	1.53**	1.271	(0.864–1.87)	0.223	1.4**			
Working part time	1.22	0.933	(0.542–1.608)	0.804	1.32*			
**Retired**	19.95**	**11.608**	**(2.192–61.469)**	**0.004**	5.49**			
**Healthcare worker**	0.81*				1.06			
**Gross Household Income**								
**Under 1333 USD per month**								
**1333 USD to 2666 USD per month**	2.16**	**1.882**	**(1.035–3.42)**	**0.038**	1.87**	**1.631**	**(0.984–2.703)**	**0.058**
**2666.67 USD to 3999.74 USD per month**	3.31**	**2.662**	**(1.477–4.8)**	**0.001**	3.61**	**2.837**	**(1.708–4.713)**	**< 0.001**
4000 USD to 5333.07 USD per month	1.6*	1.104	(0.627–1.944)	0.733	1.65**	1.148	(0.701–1.879)	0.584
**5333.34 USD to 6666.41 USD per month**	3**	**2.202**	**(1.126–4.305)**	**0.021**	4.57**	**3.508**	**(1.901–6.473)**	**< 0.001**
**6666.67 USD and over per month**	3.13**	**2.079**	**(1.184–3.648)**	**0.011**	2.71**	**1.822**	**(1.132–2.933)**	**0.013**
Don’t know/ Prefer not to say	1.62**	1.333	(0.783–2.27)	0.290	1.95**	**1.633**	**(1.026–2.599)**	**0.039**
**Perceived Current State of Health**								
**Fairly or Very poor/ Prefer not to say**								
**Very good**	4.45**	**2.361**	**(1.319–4.224)**	**0.004**	3.36**	**2.336**	**(1.399–3.9)**	**0.001**
**Fairly good**	2.68**	1.447	(0.775–2.704)	0.246	2.83**	**2.045**	**(1.171–3.571)**	**0.012**
Neither good or poor	2.45**	1.300	(0.546–3.094)	0.554	1.66*	1.233	(0.585–2.6)	0.582
**At Least One Chronic Health Conditions Currently**	0.72**				0.8**			
**Chronic Condition in Members of the Household or Those Under Your Care**	0.94				0.97			
**At Least One Respiratory/ Cold/ Flu-like Symptom**	0.43**	**0.545**	**(0.412–0.721)**	**< 0.001**	0.5**	**0.591**	**(0.464–0.752)**	**< 0.001**

*p-value < 0.20; ** *p*-value < 0.05. The first categories are the references. . OR^1^: odds ratio estimate by univariable logistic model. *OR* odds ratio estimate by multivariable logistic model

### Personal experience of problems associated with COVID-19

More than half the respondents (54%) experienced/witnessed spreading of misinformation about COVID-19 and only 4% experienced or witnessed violence in relation to COVID-19 (See Additional file 2).

### Temporal differences

During the lockdown period (21 April-22 May), 1767 respondents completed the survey, and in the post lockdown period (31 May-21 June) 606 completed the survey. There was a significant difference in anxiety during lockdown compared to post lockdown (10% vs 14%, *P* = 0.03). There was no significant difference in the adoption of preventive measures; however, the perceived effectiveness of social distancing was slightly higher in the post lockdown period compared to lockdown (99.8% vs. 98.4%, *P =* 0.02). As for hygiene practices’ perceived effectiveness, there was a slightly significant difference between the two periods with a slight increase in the post lockdown period. There was no significant difference in respondents’ ability and willingness to self-isolate between the two periods.

## Discussion

This study provides important insights into risk perception and adoption of preventive measures in a population of 2393 Saudi adults, during the lockdown and post lockdown periods in the early months of the COVID-19 pandemic. Most respondents reported that they were worried about the spread of COVID-19 and believed that if they were infected it would be either a moderate infection that requires bed rest or a severe one. Around 11% reported high anxiety levels in 14 days prior to participating in the survey. The level of anxiety post-lockdown was higher than that during the lockdown period, accompanied with an increase in the perceived effectiveness of hygiene practices and social distancing to reduce the chances of COVID-19 infection. Washing hands frequently with soap and water and avoiding crowds and social events were reported as the most adopted measures to protect oneself. While protecting others involved mostly covering one’s nose and mouth while sneezing or coughing rather than wearing a mask.

### Comparison with similar studies in existing literature

This study reported high levels of worry about the pandemic. This finding was consistent with the two studies using the same survey tool; one in the UK (*N* = 2108 respondents) [27, 28] and another in HK (*N* = 1715 respondents) [17]. Unlike the study based in the UK, but similar to the study based in HK, the perceived effectiveness of preventive measures and their adoption was high in this study. This might reflect the experience Saudi Arabia and HK had with the previous epidemics of SARS [33] and MERS [34], respectively. Also, the first case in Saudi Arabia was reported later than these countries and the survey was distributed a month later, so respondents had the opportunity to learn the positive experiences of other countries with high adoption of preventive measures through news and other forms of media.

The most common source of information in the UK population was television [27, 28]. In HK, social media platforms and websites (both official and unofficial) were used [17]. This study found that official websites and their social media platforms were commonly used to seek information about Covid-19 in Saudi Arabia. Adoption of social distancing measures was higher in Saudi Arabia (98%) compared to the UK (45.2%) [27, 28] and HK (range: 39–93%) [17]. The greater use of official websites and level of adoption of social distancing measures might be explained by the differences in context, timing and duration of data collection. In Saudi Arabi, the survey was available for almost two months during lockdown and post lockdown; additionally, the government used a transparent approach by having a large media presence and awareness messages provided by Saudi Arabia’s Ministry of Health on a daily basis through their scheduled media conferences [4, 35]. The effect of their approach is clear in this study with official websites and their associated social media outlets being used as the main source of information and perceived as the most reliable source of information by the majority of respondents. That might reflect trust in the government, which was reported by another cross-sectional study that assessed Saudi Arabian population’s trust and compliance with measures enforced by the government to prevent or reduce COVID-19 transmission during the early phase of the pandemic and found high levels of trust [36].

Despite the fact that the elderly are more vulnerable to COVID-19, the adoption of hygiene practices and social distancing was lower (in both adjusted and unadjusted models) amongst those aged 65 years and older compared to the youngest age group in this study. This finding contradicts the findings of the study in the UK, where social distancing was adopted more amongst those aged 70 years and older [27, 28]. This association between age and adoption of preventive measures has been explored in other studies and it showed inconsistencies across different countries and during different pandemics [28, 33, 37, 38]. In addition, perceived susceptibility levels were also lower among this age group, and that was similar to a survey conducted in Germany where the elderly reported lower risk perception [39]. This might be related to optimism bias where people expect better results than reality [40] (i.e. in the case of Saudi Arabia, there might be a belief that the risk is low if they are practising some degree of preventive measures). Moreover, in Saudi Arabia, religious beliefs tend to have a stronger effect on elderly health behaviors and perceptions [7]. As for the differences in gender, females tend to have higher perceived susceptibility and adoption of preventive measures compared to males across studies [17, 33, 37, 38, 41, 42]. Females also had higher levels of anxiety compared to males, which was also measured in another study conducted in Saudi Arabia during the pandemic [43]. The same study showed that those practising social distancing or hygiene practices were less likely to report anxiety, but this was not seen in the present study [43]. Even for certain older age groups who had lower anxiety levels compared to the younger age groups, they were not practising preventive measures.

Respondents in the higher income groups were less likely to worry about the pandemic, as they were more likely to be educated and less likely to be affected by the impact of COVID-19 pandemic in terms of employment and household finance compared to those in the lower income group due to existing inequalities that were heightened by the pandemic [44, 45]. The difference in perceived susceptibility and severity between the higher and lower income groups might be due to the former having better access to accurate/reliable sources of information while the latter group are more likely to be exposed to misinformation about COVID-19 [46].

### Strengths and limitations

One of the key strengths of this study is the use of an online method to collect data resulting in a large sample that exceeded the required sample size. In addition, temporal changes were investigated as the pandemic progressed and the government’s suppressive measures changed. The survey tool used in this study was also applied in other international research groups, which may enable cross-country comparisons. The translation process followed the WHO guidelines for translating instruments [29] and used quantitative and qualitative methods to ensure a rigorous process. As for the limitations, the study design does not allow for causal inferences. Most of the participants were from urban areas which might have affected generlisability of results, although most of the COVID-19 cases were located in Riyadh and Mecca (both large urban cities). The online method might have excluded an important group of the population (the elderly, people with learning disabilities or those without access to the internet), who might have been equally or more vulnerable to COVID-19, also, self-reported data may be a possible source of error. Response bias might be an issue, this was reduced through making the survey anonymous and participation voluntary. Furthermore, the sample was obtained through convenience sampling and contained people from a higher socio-economic status, which might potentially exclude the deprived socio-economic groups and other groups that did not or were unwilling to fill the survey.

### Implications for policy

This study has certain implications for public health and health policy. The most prominent one is the trust that people have in the government recommendations in Saudi Arabia. This was highlighted when the majority of the respondents reported official government social media accounts and websites as their first trustworthy source of information, and the reason for adherence or adoption of preventive measures was based on government recommendations; other cross-sectional studies showed similar favorable attitudes toward the government during the early phase of the pandemic [36, 47]. The willingness of the public to report their symptoms to health authorities and the high rate of preventive measures adoption might be to some degree influenced by government efforts. Social media and websites of health authorities were effective means of spreading key Covid-19 related messages and as they were commonly used by many different population groups in Saudi Arabia. These communication channels should be used when promoting non-pharmaceutical interventions and Covid-19 vaccinations or treatment when available [48–50].

The temporal differences in anxiety levels between lockdown and post lockdown might indicate a need for well-being intervention programs and a focus on designing health awareness messages that do not raise anxiety to the degree that could increase the burden of poor mental health associated with the pandemic worldwide [43, 51–53]. Furthermore, the heighted anxiety and poorer mental health caused by the pandemic might need to be further addressed through increased universal and targeted access to mental health support and services at national level.

Another point to consider is how to facilitate the process of self-isolation which is currently recommended for those with mild COVID-19 symptoms or those who were in contact with a COVID-19 case in Saudi Arabia [3]. There are certain factors that were associated with a decrease in willingness or/and ability to self-isolate that need to be targeted. The first one was having at least one flu symptom in the last 14 days of filling the survey, people who had symptoms were less likely to be able or willing to self-isolate; that might have reflected their own experience. The second involves those with the lowest category of income compared to others. Those two groups need to be targeted to facilitate their adoption of self-isolation as they are more likely to be vulnerable to COVID-19 [1, 3, 17, 28]. While taking into consideration the most common worries with regard to self-isolation, e.g. inability to provide food and medication or inability to self-isolate from family members or those living in the same household. Interventions or programs to provide practical support e.g. delivering food and medication to those who are self-isolating especially those with no help available from friends and families or are economically disadvantaged, need to be available. Examples of such services may include help with grocery shopping, collecting medication from pharmacies, and befriending services for people who suffer loneliness.

Public health officials also need to use strategies for identifying and communicating with at-risk populations (specifically the elderly) through site visits to nursing homes, senior citizens centers, and similar settings hosting this at-risk group. They also need to prioritize research in areas of elderly COVID-19 perceptions, particularly around barriers and facilitators to adherence to preventive measures.

### Implications for research

This study showed the potential to further investigate the effect of socio-demographic characteristics on the population’s adoption of preventive measures and risk perception, and the role of context across countries. For Saudi Arabia specifically, research interest during COVID 19 is oriented more toward epidemiology and understanding the disease’s pathway and treatment. However, it is important to understand why older population and those with lower socio-economic status have lower risk perception and adoption of preventive measures compared to the younger population. By understanding their perceptions and barriers, interventions can be developed for these groups informed by evidence. And understanding how people in general perceive risk of the disease and react to system and individual level preventive measures for the control of future outbreaks is urgently needed and can be gained through the use of qualitative studies [19].

## Conclusion

During an emerging pandemic, there is a great reliance on the public to adopt and practice preventive measures in order to stop the spread of the virus, particularly when pharmaceutical interventions are not yet available on a large scale. Understanding the factors that influence public’s response can assist in developing targeted interventions to facilitate adoption of preventive measures. By targeting specific groups through either providing appropriate resources or directing tailored interventions to change people’s behavior, it can help in increasing adoption of preventive measures and reducing COVID-19 related morbidity and mortality rate.

## Supplementary Information

**Additional file 1.**

**Additional file 2.**

## Data Availability

The datasets used and/or analysed during the current study are available from the corresponding author on reasonable request.

## Abbreviations

WHO: World Health Organization
UK: United Kingdom
HK: Hong Kong
AR: Adjusted residuals
OR: Odds ratios
COVID-19: Coronavirus Disease-19
USD: US Dollars
