# Supplementary material for: Perceptions towards COVID-19 and adoption of preventive measures among the public in Saudi Arabia: a cross sectional study
Source: BMC Public Health. 2021 Jun 29;21:1251. doi: 10.1186/s12889-021-11223-8 (PMC8240080; doi:10.1186/s12889-021-11223-8)
Supplement: Supplementary file 1 — Additional file 1. [file 12889_2021_11223_MOESM1_ESM.pdf]

COVID-19 in Saudi Arabia: A population Survey

We would like to invite you to take part in this survey conducted by a research team from King Saud University.

This study has been approved by the University's Ethics Committee.

The survey aims to understand people's risk perception and attitudes towards the novel coronavirus disease (COVID-19). Your responses will provide important insights, which could help inform current and future control measures to interrupt and delay transmission of the virus in the Kingdom of Saudi Arabia (KSA).

Any information given will be treated confidentially and only used for research purposes.

For any question regarding the research, contact Dr.Ghadah Alkhaldi (ghalkhaldi@ksu.edu.sa)

If you agree to participate and you are 18 years old and above, please click next.

Section I: General Information

1. Which age group do you belong to? *

- ◯ 18-24
- ◯ 25- 34
- ◯ 35 -44
- ◯ 45 -54
- ◯ 55 -64
- ◯ 65 -74
- ◯ 75 years old and above

2. What is your gender? *

- ◯ Male
- ◯ Female

3. Are you currently pregnant? *

- ◯ Yes, I am
- ◯ No, I am not

4. What is your marital status? *

- ◯ Married
- ◯ Separated/ Divorced
- ◯ Widowed
- ◯ Never married

5. In the past 7 days, which area have you mainly lived in? *

- ◯ Makkah
- ◯ Riyadh
- ◯ Asir
- ◯ Jawf
- ◯ Northern Borders
- ◯ Bahah
- ◯ Madinah

- Ha'il
- Najran
- Qasim
- Tabuk
- Jizan
- Eastern Province
- Other, please specify

6. What is the highest educational or work-related qualification you have? *

- Read and Write
- Primary
- Intermediate
- Secondary/Equivalent
- Pre-Univ.Diploma
- University
- High Diploma
- Master
- PhD
- Prefer not to say

7. What is your nationality? *

- Saudi
- Non-Saudi
- Prefer not to say

8. Which of these applies to you? *

- Working full time (30 or more hours per week)
- Working part time (8-29 hours a week)
- Working part time (Less than 8 hours a week)
- Full time student
- Retired
- Unemployed
- Not working
- Other

9. Are you a healthcare worker (e.g. doctor, nurse, paramedic etc.) or studying a medical course or an intern? *

- Yes, I am
- No, I am not

10. Gross HOUSEHOLD income is the combined income of all those earners in a household from all sources, including wages, salaries, or rents. What is your gross household income? *

- under 5,000 SR per month
- SR 5,000 SR to 9,999 per month
- SR 10,000 to SR 14,999 per month
- SR 15,000 to SR 19,999 per month
- SR 20,000 to SR 24,999 per month
- SR 25,000 and over per month

- Don't know
- Prefer not to say

11. Which, if any, of the following people do you have any caring responsibilities for? (Please select all that apply. If your answer is not provided in the list shown, please type it in the 'Other' box) *

- Child(ren) aged under 5
- Child(ren) aged 5 to 16
- Elderly relative/ dependent
- Disabled dependent
- Not applicable
- Prefer not to say
- Other, please specify

12. In general, how good or poor, if at all, would you say your current state of health is (i.e. mental and physical)? *

- Very good
- Fairly good
- Neither good or poor
- Fairly poor
- Very poor
- Prefer not to say

13. Which, if any, of the following chronic health conditions do you currently have/ have you had in the last 6 months (i.e. since October 2019)? (Please select all that apply. If you do not currently have a chronic health condition, please select the 'Not applicable' option) *

- Eye conditions (e.g. glaucoma, cataract, blindness, etc.)
- Ear, nose and/ or throat condition (e.g. allergic rhinitis, deaf, whizzing, etc.)
- Cancer
- Epilepsy/ seizure
- Stroke
- Hypertension
- Heart disease
- Asthma
- Emphysema, bronchitis, bronchiectasis
- Tuberculosis
- Thyroid glands disease
- Diabetes mellitus (i.e. Type 1 or Type 2)
- Hyperlipidaemia
- Kidney condition (e.g. kidney failure, nephritis, etc.)
- Liver condition (e.g. Hepatitis B/C, cirrhosis, etc.)
- Bowel condition (e.g. gastric ulcer, etc.)
- Anaemia
- Genetic blood disorders (e.g. thalassemia, haemophilia, etc.)
- Skeletomuscular disorders (e.g. arthritis, rheumatism, gout, osteoporosis, etc.)
- Autoimmune disorder (e.g. systemic lupus erythematosus, rheumatoid arthritis, etc.)
- Skin condition (e.g. eczema, psoriasis, etc.)
- Depression
- Anxiety
- Schizophrenia
- Not applicable - I do not currently have a chronic health condition
- Prefer not to say
- Other, please specify

14. Does anyone else in your household, or who you have caring responsibilities for, have any chronic health conditions? *

- Yes, they do
- No, they don't
- Don’t know
- Prefer not to say

15. Thinking about the past 14 days...
Which, if any, of the following respiratory/ cold/ flu-like symptoms have you experienced? (Please select all that apply. If you have not experienced any respiratory/ cold/ flu-like symptoms in the past 14 days, please select the 'Not applicable' option) *

- Persistent fever (i.e. body temperature over 38°C for at least one day)
- Shivering
- Headache
- Muscle pain
- Cough
- Difficulty in breathing or shortness of breath
- Dizziness
- Runny nose
- Sore throat
- Not applicable - I have not experienced any respiratory, cold or flu-like symptoms in the past 14 days
- Prefer not to say
- Other, please specify

Section II: Measuring the impact of the current situation on your psychological state

Thinking about the past week...To what extent, if at all, have you been feeling the following?

16. "I feel tense or 'wound up'." *

- Most of the time
- A lot of the time
- From time to time, occasionally
- Not at all

17. "I get a sort of frightened feeling as if something awful is about to happen." *

- Very definitely and quite badly
- Yes, but not too badly
- A little, but it doesn't worry me
- Not at all

18. "Worrying thoughts go through my mind." *

- A great deal of the time
- A lot of the time
- From time to time, but not too often
- Only occasionally

19. "I can sit at ease and feel relaxed." *

- Definitely
- Usually
- Not often
- Not at all

20. "I get a sort of frightened feeling like 'butterflies' in the stomach." *

- Very often
- Quite often
- Occasionally
- Not at all

21. "I feel restless as I have to be on the move." *

- Very much indeed
- Quite a lot
- Not very much
- Not at all

22. "I get sudden feelings of panic." *

- Very often indeed
- Quite often
- Not very often
- Not at all

23. "I still enjoy the things I used to enjoy." *

- Definitely as much
- Not quite as much
- Only a little
- Hardly at all

24. "I can laugh and see the funny side of things." *

- As much as I always could
- Not quite as much now
- Definitely not so much now
- Not at all

25. "I feel cheerful." *

- Most of the time
- Sometimes
- Not often
- Not at all

26. "I feel as if I am slowed down." *

- Nearly all the time
- Very often
- Sometimes
- Not at all

27. "I have lost interest in my appearance." *

- Definitely
- I don't take as much care as I should
- I may not take quite as much care
- I take just as much care as ever

28. "I look forward with enjoyment to things." *

- As much as I ever did
- Rather less than I used to
- Definitely less than I used to
- Hardly at all

29. "I can enjoy a good book or radio or TV programme." *

- Often
- Sometimes
- Not often
- Very seldom

Section III: Perceptions and attitude regarding COVID-19

The 2019 coronavirus, otherwise known as COVID-19, is an infectious disease first identified in the city of Wuhan, capital of Hubei province in China. Infections have since been reported around the world. Symptoms include fever, coughing and breathing difficulties.

30. In general, how worried, if at all, are you about the current coronavirus (i.e. COVID-19) outbreak in KSA? *

- Very worried
- Fairly worried
- Not very worried
- Not at all worried
- Don’t know

31. Thinking about the past 14 days...
Have you personally been tested for coronavirus (i.e. COVID-19)? (Please select the option that best applies) *

- Yes, and I tested positive for coronavirus
- Yes, and I tested negative for coronavirus
- Yes, and I have not received my results from the test yet
- No, I have not
- Prefer not to say

32. Under the KSA government's current preventive measures, how likely or unlikely do you think it is you will be infected with the coronavirus (COVID-19) at any point in the future? *

- Very likely
- Fairly likely
- Neither likely or unlikely
- Fairly unlikely
- Very unlikely
- Don’t know

33. For the following question, please imagine you were infected with coronavirus (i.e. COVID-19).
Which of the following do you think would best apply? *

- I would expect it to be life-threatening
- I would expect it to be severe (e.g. may need care and treatment in hospital)
- I would expect it to be moderate (e.g. may need self-care and rest in bed)
- I would expect it to be mild (e.g. can go about daily tasks normally)
- I would expect to have no symptoms
- Don’t know

34. From which, if any, of the following sources are you currently getting information about the coronavirus (i.e. COVID-19)? (Please select all that apply. If you are not getting any information about coronavirus, please select the 'Not applicable' option) *

- [ ] Newspaper(s) (i.e. in print or online)
- [ ] Magazine(s) (i.e. in print or online)
- [ ] Radio
- [ ] Television, excluding streaming services
- [ ] Streaming services (e.g. Netflix, Amazon Prime, etc.)
- [ ] Official websites and their social media outlets(e.g. government, MOH, Wegaya, World Health Organization, etc.)
- [ ] Unofficial websites (i.e. not authorised or acknowledged by the government or an organisation in authority)
- [ ] Social media platforms (e.g. YouTube, WhatsApp, Twitter, Facebook, Instagram)
- [ ] My doctor
- [ ] Other healthcare professional, excluding my doctor
- [ ] My family or friends
- [ ] Work/ school/ college communications
- [ ] Not applicable - I am not getting any information about coronavirus (i.e. COVID-19)
- [ ] Other, please specify

35. Which, if any, of the following types of information about the coronavirus (i.e. COVID-19) would you like to receive from a trusted source? (Please select all that apply. If you would not like to receive any information about the coronavirus, please select the 'Not applicable' option) *

- [ ] Latest research explaining what is known about coronavirus
- [ ] Common signs and symptoms of infection with coronavirus
- [ ] Less common signs and symptoms of infection with coronavirus
- [ ] How to know if I am infected with coronavirus
- [ ] What to do if I am infected with coronavirus
- [ ] How coronavirus is spread/ transmitted
- [ ] What to do to protect myself from getting infected with coronavirus
- [ ] Impact of coronavirus on high-risk population groups
- [ ] The current number of infected cases in KSA
- [ ] The current distribution of cases in KSA
- [ ] The current risk of getting infected in KSA
- [ ] The latest intervention measures against coronavirus put in place by the KSA government
- [ ] The latest intervention measures against coronavirus put in place by other countries’ governments
- [ ] The latest intervention measures against coronavirus put in place by international organisations (e.g. World Health Organisation, etc.)
- [ ] Not applicable - I don't want to receive any information about the coronavirus
- [ ] Other, please specify

36. In general, how reliable or unreliable, if at all, do you think each of the following information sources for information on coronavirus are? (Please select one option on each row) *

	Very reliable	Fairly reliable	Neither reliable or unreliable	Fairly unreliable	Very unreliable	Don’t know

Films/ TV shows on streaming services (e.g. Netflix, Amazon Prime, etc.)	○	○	○	○	○	○
Official websites and their social media outlets (e.g. government, MOH, Wegaya, etc.)	○	○	○	○	○	○
Unofficial websites (i.e. not authorised or acknowledged by the government or an organisation in authority)	○	○	○	○	○	○
Social media platforms (e.g. Whatsapp, Twitter, Facebook, Instagram, YouTube, etc.)	○	○	○	○	○	○
My doctor	○	○	○	○	○	○
Other healthcare professionals, excluding my doctor	○	○	○	○	○	○
My family and/ or friends	○	○	○	○	○	○
Work/ school/ college communications	○	○	○	○	○	○

37. How likely or unlikely, if at all, do you think it is that the coronavirus (i.e. COVID-19) is transmitted through each of the following? (Please select one option on each row) *

	Very likely	Fairly likely	Neither likely or unlikely	Fairly unlikely	Very unlikely	Don't know
Having a face-to-face conversation with someone who has coronavirus but no symptoms (without physical contact)	○	○	○	○	○	○
Having a face-to-face conversation with someone who has coronavirus with symptoms (without physical contact)	○	○	○	○	○	○
Having physical contact with someone who has coronavirus but no symptoms	○	○	○	○	○	○
Having physical contact with someone with coronavirus who has symptoms	○	○	○	○	○	○
Being in close contact (i.e. within 2 meters) to someone who has coronavirus, when they cough or sneeze	○	○	○	○	○	○
Being further away (i.e. further than 2 meters away) to someone who has coronavirus, when they cough or sneeze	○	○	○	○	○	○
Contact with contaminated environments (e.g. surfaces such as lifts, ATMs, etc.)	○	○	○	○	○	○
Consumption of wild animal meat (e.g. rabbit, venison, pheasant, etc.)	○	○	○	○	○	○
Visiting public markets that sell fresh meat, fish or poultry	○	○	○	○	○	○
Consumption of seafood imported from China	○	○	○	○	○	○
Consumption/ use of products imported from China	○	○	○	○	○	○

38. For the following question, please exclude any measures that you have already taken for reasons other than coronavirus (i.e. COVID-19).

Which, if any, of the following measures have you personally taken to protect yourself and/or others from the coronavirus (i.e. COVID-19)? (Please select all that apply. If you have not taken any measures, please select the 'Not applicable' option) *

	Measures taken to protect myself	Measures taken to protect others
Worn a face mask	☐	☐
Washed hands more frequently with soap and water	☐	☐
Used hand sanitiser more regularly	☐	☐
Disinfected my home	☐	☐
Covered my nose and mouth when sneezing or coughing	☐	☐
Avoided contact with people who have a fever or respiratory symptoms (e.g. a		

cough)	☐	☐
Avoided contact with people who have been to affected areas within the last 14 days	☐	☐
Avoided going out in general	☐	☐
Avoided crowded areas	☐	☐
Avoided going to public markets that sell fresh meat, fish or poultry	☐	☐
Avoided going to hospital or other healthcare settings	☐	☐
Avoided taking public transport	☐	☐
Avoided going to work	☐	☐
Avoided going into shops and supermarkets	☐	☐
Avoided social events	☐	☐
Avoided travel to affected areas in the world	☐	☐
Avoided travel to other areas (outside KSA), regardless of whether they're affected	☐	☐
Avoided travel to other areas (inside KSA), regardless of whether they're affected	☐	☐
Moved temporarily to the countryside or a remote location	☐	☐
Don't know/ can't recall	☐	☐
Not applicable	☐	☐

39. Other measures taken to protect yourself and/or others, please specify

40. You previously mentioned that you have changed your behaviour to protect yourself and/or others from coronavirus (i.e. COVID-19)...

Which, if any, of the following was this in response to? (Please select all that apply. If your answers do not appear in the list below, please type them into the 'Other' box. If you have not taken any measures, please select the 'Not applicable' option) *

- ☐ In response to the KSA government guidance
- ☐ In response to news coverage of the outbreak
- ☐ In response to the first death to COVID-19 in KSA
- ☐ In response to the first death to COVID-19 in Europe
- ☐ In response to the first death to COVID-19 in China
- ☐ In response to news of people stock-piling
- ☐ In response to the growing number of COVID-19 cases in KSA
- ☐ In response to what is happening in other countries (e.g. Italy, Iran, etc.)
- ☐ Due to my own personal experience of the outbreak
- ☐ Don't know/ can't recall
- ☐ Not applicable- I have not taken any measures to protect myself/others
- ☐ Other, please specify

41. In general, how effective or ineffective do you think each of the following measures are in preventing the spread of coronavirus (COVID-19)? (Please select one option on each row) *

	Very effective	Fairly effective	Fairly ineffective	Very ineffective	Don't know
Wearing face masks	○	○	○	○	○
Washing hands frequently with soap and water	○	○	○	○	○

Using hand sanitiser	○	○	○	○	○
Disinfecting homes	○	○	○	○	○
Covering your nose and mouth when sneezing or coughing	○	○	○	○	○
Avoiding contact with people who have a fever or respiratory symptoms (e.g. a cough)	○	○	○	○	○
Avoiding contact with people who have been to affected areas within the last 14 days	○	○	○	○	○
Avoiding going out in general	○	○	○	○	○
Avoiding crowded areas	○	○	○	○	○
Avoiding going to public markets that sell fresh meat, fish or poultry	○	○	○	○	○
Avoiding going to hospital or other healthcare settings	○	○	○	○	○
Avoiding taking public transport	○	○	○	○	○
Avoiding going to work	○	○	○	○	○
Avoiding going to school or avoid letting your children go to school	○	○	○	○	○
Avoiding going into shops and supermarkets	○	○	○	○	○
Avoiding social events	○	○	○	○	○
Avoiding travel to affected areas in the world	○	○	○	○	○
Avoiding travel to other areas (outside KSA), regardless of whether they're affected	○	○	○	○	○
Avoiding travel to other areas (inside KSA), regardless of whether they're affected	○	○	○	○	○
Moving temporarily to the countryside or a remote location	○	○	○	○	○

42. According to the Ministry of Health, to 'self-isolate' means if you just returned from traveling abroad from certain countries or came in contact with an infected person, do not leave your home (even to buy food or essentials) or have any visitors for 14 days. This includes not going to work, or other public places, and avoiding public transport or taxis. Self-isolation is the same as voluntary quarantine.
If you were advised to do so by a healthcare professional, would you be able and willing to self-isolate? (Please select one option on each row) *

	Yes, I would	No, I wouldn't	Don't know
Able to self-isolate	○	○	○
Willing to self-isolate	○	○	○

43. For the following question, if you have not done anything to prepare for a potential self-isolation, please select the 'Not applicable' option.
Which, if any, of the following have you done in order to prepare for a potential self-isolation? (Please select all that apply. If your answer(s) do not appear in the list below, please type them into the 'Other' box) *

- ☐ Stocking up on food supplies
- ☐ Stocking up on toiletries (e.g. toilet paper, shampoo, soap, hand sanitizer, etc.)
- ☐ Stocking up on prescription medicines
- ☐ Stocking up on over-the-counter medicines (e.g. paracetamol, ibuprofen, etc.)
- ☐ Establishing remote working capabilities (e.g. sending files to home, setting up a VPN connection etc.)
- ☐ Finding alternative childcare
- ☐ Don't know/ can't recall
- ☐ Not applicable
- ☐ Other, please specify

44. For the following question, if you do not see any potential problems as you are self-isolating, please select the 'Not applicable' option.

Which, if any, of the following do you see as potential problems as you are self-isolating? (Please select all that apply. If your answer(s) do not appear in the list below, please type them into the 'Other' box) *

- ☐ It would negatively affect my mental health (e.g. I would get bored, stressed, lonely, anxious, angry, etc.)
- ☐ I would find it difficult to separate myself from others in my household (e.g. my children, housemates, etc.)
- ☐ I would find it difficult to get supplies, (e.g. food, medicines, etc.)
- ☐ I have caring responsibilities and would find it difficult to find someone to cover those (e.g. I care for a dependent child, someone with disabilities, an elderly relative, neighbour, etc.)
- ☐ It would negatively affect my social life (e.g. it would negatively impact my friendships, I would miss attending social and cultural events, etc.)
- ☐ I would experience a loss of income
- ☐ My studies or education would suffer
- ☐ Don't know
- ☐ Not applicable - I do not see any potential problems if I had to self-isolate
- ☐ Other, please specify

45. How likely or unlikely would you be to NOT report or seek help for symptoms of coronavirus to avoid self-isolation measures in each of the following situations? (Please select one option on each row) *

	Very likely	Fairly likely	Fairly unlikely	Very unlikely	Don't know
If I had mild symptoms (e.g. can go about daily tasks normally)	○	○	○	○	○
If I had moderate symptoms (e.g. may need self-care and rest in bed)	○	○	○	○	○
If I had severe symptoms (e.g. may need care and treatment in hospital)	○	○	○	○	○

46. Which, if any, of the following have you personally experienced/ witnessed in relation to coronavirus (i.e. COVID-19)? (Please select all that apply) *

- ☐ Harassment / discrimination
- ☐ Spread of misinformation about the disease or outbreak
- ☐ Racist actions
- ☐ Discrimination against a group based on background or country of origin
- ☐ Hostility towards someone taking preventative measures (e.g. wearing a face mask, refusing to shake hands)
- ☐ Violence
- ☐ Other anti-social behaviours
- ☐ None of these
- ☐ Don't know/ can't recall
- ☐ Prefer not to say

Thank you for taking part in this survey.

47. Invitation to participate in interviews:
We are conducting telephone interviews to understand people's perceptions regarding the effectiveness and feasibility of the preventive measures on a personal and community level, and barriers and facilitators to complying with preventive measures. If you are interested to participate, please provide us with your contact details. A researcher will get in touch and schedule a time and date convenient for you.

Ministry of Health information regarding the novel coronavirus disease (COVID-19) can be found here:
https://www.moh.gov.sa/CCC/FAQs/Corona/Pages/default.aspx
