# Supplementary material for: Perceptions towards COVID-19 and adoption of preventive measures among the public in Saudi Arabia: a cross sectional study
Source: BMC Public Health. 2021 Jun 29;21:1251. doi: 10.1186/s12889-021-11223-8 (PMC8240080; doi:10.1186/s12889-021-11223-8)
Supplement: Supplementary file 2 — Additional file 2. [file 12889_2021_11223_MOESM2_ESM.docx]

**Table 1. Descriptive Statistics Measuring Anxiety and Depression using HADS (Section II in the survey): absolute (n), and weighted relative (%w) frequencies.**

|  |  | ***n*** | ***%*** | ***%w*** |
| --- | --- | --- | --- | --- |
| **Anxiety** | Normal | 1744 | 72.9 | 77.2 |
|  | Borderline abnormal (borderline cases) | 320 | 13.4 | 12.1 |
|  | Abnormal (cases) | 329 | 13.7 | 10.8 |
| **Depression** | Normal | 1612 | 67.4 | 67.7 |
|  | Borderline abnormal (borderline cases) | 433 | 18.1 | 17.9 |
|  | **Abnormal (cases)** | **348** | **14.5** | **14.3** |
| **Total** |  | **2393** | **100.0** | **100.0** |

**Table 2. Descriptive Statistics Perceptions and Attitude Regarding COVID-19 (Section III in the survey): absolute (n) and relative (%) frequencies.**

|  |  | | | | | | | | | | | | | | | | | | | | ***n*** | | | | ***%*** | | | | | | | | | | ***%w*** | | | | | | | |
| --- | --- | --- | --- | --- | --- | --- | --- | --- | --- | --- | --- | --- | --- | --- | --- | --- | --- | --- | --- | --- | --- | --- | --- | --- | --- | --- | --- | --- | --- | --- | --- | --- | --- | --- | --- | --- | --- | --- | --- | --- | --- | --- |
| **In general, how worried, if at all, are you about the current coronavirus (i.e. COVID-19) outbreak in KSA?** | | | | | | | | | | | | | | | | | | | | | | | | | | | | | | | | | | |  | | | | | | | |
|  | Very worried | | | | | | | | | | | | | | | | | | | | 629 | | | | 26.3 | | | | | | | | | | 21.7 | | | | | | | |
|  | Fairly worried | | | | | | | | | | | | | | | | | | | | 1241 | | | | 51.9 | | | | | | | | | | 52.5 | | | | | | | |
|  | Not very worried | | | | | | | | | | | | | | | | | | | | 355 | | | | 14.8 | | | | | | | | | | 17.7 | | | | | | | |
|  | Not at all worried | | | | | | | | | | | | | | | | | | | | 128 | | | | 5.3 | | | | | | | | | | 6.1 | | | | | | | |
|  | Don’t know | | | | | | | | | | | | | | | | | | | | 40 | | | | 1.7 | | | | | | | | | | 2.0 | | | | | | | |
| **Thinking about the past 14 days... Have you personally been tested for coronavirus (i.e. COVID-19)? (Please select the option that best applies)** | | | | | | | | | | | | | | | | | | | | | | | | | | | | | | | | | | |  | | | | | | | |
|  | Yes, and I tested positive for coronavirus | | | | | | | | | | | | | | | | | | | | 6 | | | | 0.3 | | | | | | | | | | 0.2 | | | | | | | |
|  | Yes, and I tested negative for coronavirus | | | | | | | | | | | | | | | | | | | | 57 | | | | 2.4 | | | | | | | | | | 3.5 | | | | | | | |
|  | Yes, and I have not received my results from the test yet | | | | | | | | | | | | | | | | | | | | 10 | | | | 0.4 | | | | | | | | | | 0.5 | | | | | | | |
|  | No, I have not | | | | | | | | | | | | | | | | | | | | 2299 | | | | 96.1 | | | | | | | | | | 94.4 | | | | | | | |
|  | Prefer not to say | | | | | | | | | | | | | | | | | | | | 21 | | | | 0.9 | | | | | | | | | | 1.4 | | | | | | | |
| **Under the KSA government's current preventive measures, how likely or unlikely do you think it is you will be infected with the coronavirus (COVID-19) at any point in the future?** | | | | | | | | | | | | | | | | | | | | | | | | | | | | | | | | | | |  | | | | | | | |
|  | Very likely | | | | | | | | | | | | | | | | | | | | 98 | | | | 4.1 | | | | | | | | | | 4.1 | | | | | | | |
|  | Fairly likely | | | | | | | | | | | | | | | | | | | | 461 | | | | 19.3 | | | | | | | | | | 20.0 | | | | | | | |
|  | Neither likely or unlikely | | | | | | | | | | | | | | | | | | | | 619 | | | | 25.9 | | | | | | | | | | 25.2 | | | | | | | |
|  | Fairly unlikely | | | | | | | | | | | | | | | | | | | | 494 | | | | 20.7 | | | | | | | | | | 20.2 | | | | | | | |
|  | Very unlikely | | | | | | | | | | | | | | | | | | | | 285 | | | | 11.9 | | | | | | | | | | 11.7 | | | | | | | |
|  | Don’t know | | | | | | | | | | | | | | | | | | | | 430 | | | | 18.0 | | | | | | | | | | 18.9 | | | | | | | |
| **For the following question, please imagine you were infected with coronavirus (i.e. COVID-19). Which of the following do you think would best apply?** | | | | | | | | | | | | | | | | | | | | | | | | | | | | | | | | | | |  | | | | | | | |
|  | I would expect it to be life-threatening | | | | | | | | | | | | | | | | | | | | 47 | | | | 2.0 | | | | | | | | | | 2.0 | | | | | | | |
|  | I would expect it to be severe (e.g. may need care and treatment in hospital) | | | | | | | | | | | | | | | | | | | | 321 | | | | 13.4 | | | | | | | | | | 12.4 | | | | | | | |
|  | I would expect it to be moderate (e.g. may need self-care and rest in bed) | | | | | | | | | | | | | | | | | | | | 916 | | | | 38.4 | | | | | | | | | | 37.2 | | | | | | | |
|  | I would expect it to be mild (e.g. can go about daily tasks normally) | | | | | | | | | | | | | | | | | | | | 470 | | | | 19.7 | | | | | | | | | | 18.4 | | | | | | | |
|  | I would expect to have no symptoms | | | | | | | | | | | | | | | | | | | | 183 | | | | 7.7 | | | | | | | | | | 7.5 | | | | | | | |
|  | Don’t know | | | | | | | | | | | | | | | | | | | | 450 | | | | 18.9 | | | | | | | | | | 22.4 | | | | | | | |
| **From which, if any, of the following sources are you currently getting information about the coronavirus (i.e. COVID-19)? (Please select all that apply** | | | | | | | | | | | | | | | | | | | | | | | | | | | | | | | | | | |  | | | | | | | |
|  | Newspaper(s) | | | | | | | | | | | | | | | | | | | | 353 | | | | 14.8 | | | | | | | | | | 16.5 | | | | | | | |
|  | Magazine(s) | | | | | | | | | | | | | | | | | | | | 69 | | | | 2.9 | | | | | | | | | | 4.4 | | | | | | | |
|  | Radio | | | | | | | | | | | | | | | | | | | | 61 | | | | 2.5 | | | | | | | | | | 3.0 | | | | | | | |
|  | Television, excluding streaming services | | | | | | | | | | | | | | | | | | | | 792 | | | | 33.1 | | | | | | | | | | 36.8 | | | | | | | |
|  | Streaming services | | | | | | | | | | | | | | | | | | | | 64 | | | | 2.7 | | | | | | | | | | 3.7 | | | | | | | |
|  | Official websites and their social media outlets | | | | | | | | | | | | | | | | | | | | 1742 | | | | 72.8 | | | | | | | | | | 71.8 | | | | | | | |
|  | Unofficial websites | | | | | | | | | | | | | | | | | | | | 94 | | | | 3.9 | | | | | | | | | | 5.2 | | | | | | | |
|  | Social media platforms | | | | | | | | | | | | | | | | | | | | 1227 | | | | 51.3 | | | | | | | | | | 48.4 | | | | | | | |
|  | My doctor | | | | | | | | | | | | | | | | | | | | 57 | | | | 2.4 | | | | | | | | | | 2.9 | | | | | | | |
|  | Other healthcare professional, excluding my doctor | | | | | | | | | | | | | | | | | | | | 79 | | | | 3.3 | | | | | | | | | | 3.3 | | | | | | | |
|  | My family or friends | | | | | | | | | | | | | | | | | | | | 536 | | | | 22.4 | | | | | | | | | | 18.2 | | | | | | | |
|  | Work/ school/ college communications | | | | | | | | | | | | | | | | | | | | 292 | | | | 12.2 | | | | | | | | | | 12.4 | | | | | | | |
|  | Not applicable | | | | | | | | | | | | | | | | | | | | 71 | | | | 3.0 | | | | | | | | | | 2.9 | | | | | | | |
|  | Other | | | | | | | | | | | | | | | | | | | | 31 | | | | 1.3 | | | | | | | | | | 1.7 | | | | | | | |
| **Which, if any, of the following types of information about the coronavirus (i.e. COVID-19) would you like to receive from a trusted source? (Please select all that apply).** | | | | | | | | | | | | | | | | | | | | | | | | | | | | | | | | | | |  | | | | | | | |
|  | Latest research explaining what is known about coronavirus | | | | | | | | | | | | | | | | | | | | 1354 | | | | 56.6 | | | | | | | | | | 56.5 | | | | | | | |
|  | Common signs and symptoms of infection with coronavirus | | | | | | | | | | | | | | | | | | | | 952 | | | | 39.8 | | | | | | | | | | 39.1 | | | | | | | |
|  | Less common signs and symptoms of infection with coronavirus | | | | | | | | | | | | | | | | | | | | 733 | | | | 30.6 | | | | | | | | | | 28.8 | | | | | | | |
|  | How to know if I am infected with coronavirus | | | | | | | | | | | | | | | | | | | | 903 | | | | 37.7 | | | | | | | | | | 36.6 | | | | | | | |
|  | What to do if I am infected with coronavirus | | | | | | | | | | | | | | | | | | | | 934 | | | | 39.0 | | | | | | | | | | 37.8 | | | | | | | |
|  | How coronavirus is spread/ transmitted | | | | | | | | | | | | | | | | | | | | 797 | | | | 33.3 | | | | | | | | | | 33.4 | | | | | | | |
|  | What to do to protect myself from getting infected with coronavirus | | | | | | | | | | | | | | | | | | | | 951 | | | | 39.7 | | | | | | | | | | 40.2 | | | | | | | |
|  | Impact of coronavirus on high-risk population groups | | | | | | | | | | | | | | | | | | | | 631 | | | | 26.4 | | | | | | | | | | 26.5 | | | | | | | |
|  | The current number of infected cases in KSA | | | | | | | | | | | | | | | | | | | | 1155 | | | | 48.3 | | | | | | | | | | 48.4 | | | | | | | |
|  | The current distribution of cases in KSA | | | | | | | | | | | | | | | | | | | | 1004 | | | | 42.0 | | | | | | | | | | 42.9 | | | | | | | |
|  | The current risk of getting infected in KSA | | | | | | | | | | | | | | | | | | | | 747 | | | | 31.2 | | | | | | | | | | 31.3 | | | | | | | |
|  | The latest intervention measures against coronavirus put in place by the KSA government | | | | | | | | | | | | | | | | | | | | 1054 | | | | 44.0 | | | | | | | | | | 43.3 | | | | | | | |
|  | The latest intervention measures against coronavirus put in place by other countries’ governments | | | | | | | | | | | | | | | | | | | | 597 | | | | 24.9 | | | | | | | | | | 25.1 | | | | | | | |
|  | The latest intervention measures against coronavirus put in place by international organisations (e.g. World Health Organisation, etc.) | | | | | | | | | | | | | | | | | | | | 596 | | | | 24.9 | | | | | | | | | | 24.0 | | | | | | | |
|  | Not applicable | | | | | | | | | | | | | | | | | | | | 282 | | | | 11.8 | | | | | | | | | | 11.2 | | | | | | | |
|  | Other | | | | | | | | | | | | | | | | | | | | 17 | | | | .7 | | | | | | | | | | 0.6 | | | | | | | |
| **In general, how reliable or unreliable, if at all, do you think each of the following information sources for information on coronavirus are? (Please select one option on each row)** | | | | | | | | | | | | | | | | | | | | | | | | | | | | | | | | | | | | | | | | | |  |
|  | | |  | | | *Very reliable* | | | | *Fairly reliable* | | | | | *Neither reliable or unreliable* | | | *Fairly unreliable* | | | | | | | *Very unreliable* | | | | | | | | | | *Don’t know* | | | | | | |  |
| Films/ TV shows on streaming services | | | n | | | 87 | | | | 240 | | | | | 418 | | | 474 | | | | | | | 867 | | | | | | | | | | | | 307 | | | | |  |
|  | | | % | | | 3.6 | | | | 10.0 | | | | | 17.5 | | | 19.8 | | | | | | | 36.2 | | | | | | | | | | 12.8 | | | | | | |  |
|  | | | %w | | | 4.8 | | | | 11.7 | | | | | 19.1 | | | 17.9 | | | | | | | 32.4 | | | | | | | | | | 14.1 | | | | | | |  |
| Official websites and their social media outlets | | | n | | | 1754 | | | | 444 | | | | | 105 | | | 41 | | | | | | | 19 | | | | | | | | | | 30 | | | | | | |  |
|  | | | % | | | 73.3 | | | | 18.6 | | | | | 4.4 | | | 1.7 | | | | | | | 0.8 | | | | | | | | | | 1.3 | | | | | | |  |
|  | | | %w | | | 69.9 | | | | 20.5 | | | | | 5.4 | | | 1.8 | | | | | | | 1.0 | | | | | | | | | | 1.5 | | | | | | |  |
| Unofficial websites | | | n | | | 25 | | | | 125 | | | | | 313 | | | 472 | | | | | | | 1239 | | | | | | | | | | 219 | | | | | | |  |
|  | | | % | | | 1.0 | | | | 5.2 | | | | | 13.1 | | | 19.7 | | | | | | | 51.8 | | | | | | | | | | 9.2 | | | | | | |  |
|  | | | %w | | | 1.9 | | | | 5.6 | | | | | 14.4 | | | 19.9 | | | | | | | 48.2 | | | | | | | | | | 10.1 | | | | | | |  |
| Social media platforms | | | n | | | 96 | | | | 421 | | | | | 497 | | | 593 | | | | | | | 678 | | | | | | | | | | 108 | | | | | | |  |
|  | | | % | | | 4.0 | | | | 17.6 | | | | | 20.8 | | | 24.8 | | | | | | | 28.3 | | | | | | | | | | 4.5 | | | | | | |  |
|  | | | %w | | | 4.6 | | | | 17.0 | | | | | 21.3 | | | 23.4 | | | | | | | 28.6 | | | | | | | | | | 5.0 | | | | | | |  |
| My doctor | | | n | | | 752 | | | | 794 | | | | | 431 | | | 125 | | | | | | | 68 | | | | | | | | | | 223 | | | | | | |  |
|  | | | % | | | 31.4 | | | | 33.2 | | | | | 18.0 | | | 5.2 | | | | | | | 2.8 | | | | | | | | | | 9.3 | | | | | | |  |
|  | | | %w | | | 33.7 | | | | 31.2 | | | | | 17.1 | | | 4.9 | | | | | | | 2.9 | | | | | | | | | | 10.3 | | | | | | |  |
| Other healthcare professionals, excluding my doctor | | | n | | | 486 | | | | 810 | | | | | 570 | | | 183 | | | | | | | 117 | | | | | | | | | | 227 | | | | | | |  |
|  | | | % | | | 20.3 | | | | 33.8 | | | | | 23.8 | | | 7.6 | | | | | | | 4.9 | | | | | | | | | | 9.5 | | | | | | |  |
|  | | | %w | | | 20.4 | | | | 34.0 | | | | | 22.7 | | | 7.8 | | | | | | | 5.2 | | | | | | | | | | 9.9 | | | | | | |  |
| My family and/ or friends | | | n | | | 90 | | | | 377 | | | | | 780 | | | 550 | | | | | | | 438 | | | | | | | | | | 158 | | | | | | |  |
|  | | | % | | | 3.8 | | | | 15.8 | | | | | 32.6 | | | 23.0 | | | | | | | 18.3 | | | | | | | | | | 6.6 | | | | | | |  |
|  | | | %w | | | 4.6 | | | | 16.9 | | | | | 32.4 | | | 21.5 | | | | | | | 17.4 | | | | | | | | | | 7.3 | | | | | | |  |
| Work/ school/ college communications | | | n | | | 220 | | | | 521 | | | | | 670 | | | 402 | | | | | | | 370 | | | | | | | | | | 210 | | | | | | |  |
|  | | | % | | | 9.2 | | | | 21.8 | | | | | 28.0 | | | 16.8 | | | | | | | 15.5 | | | | | | | | | | 8.8 | | | | | | |  |
|  | | | %w | | | 10.4 | | | | 22.0 | | | | | 28.7 | | | 15.4 | | | | | | | 14.3 | | | | | | | | | | 9.1 | | | | | | |  |
| **How likely or unlikely, if at all, do you think it is that the coronavirus (i.e. COVID-19) is transmitted through each of the following? (Please select one option on each row)** | | | | | | | | | | | | | | | | | | | | | | | | | | | | | | | | | | | | | | | | | |  |
|  | | | | | | | |  | *Very likely* | | | *Fairly likely* | | | | *Neither likely or unlikely* | | | | *Fairly unlikely* | | | | | | *Very unlikely* | | | | | | | | | | *Don’t know* | | | | | |  |
| Having a face-to-face conversation  with someone who has coronavirus  but no symptoms  (without physical contact) | | | | | | | | n | 1095 | | | 923 | | | | 145 | | | | 128 | | | | | | 42 | | | | | | | | | | 60 | | | | | |  |
|  | | | | | | | | % | 45.8 | | | 38.6 | | | | 6.1 | | | | 5.3 | | | | | | 1.8 | | | | | | | | | | 2.5 | | | | | |  |
|  | | | | | | | | %w | 44.5 | | | 39.0 | | | | 6.2 | | | | 5.7 | | | | | | 1.8 | | | | | | | | | | 2.8 | | | | | |  |
| Having a face-to-face conversation  with someone who has coronavirus  with symptoms (without physical contact) | | | | | | | | n | 1455 | | | 671 | | | | 108 | | | | 75 | | | | | | 32 | | | | | | | | | | 52 | | | | | |  |
|  | | | | | | | | % | 60.8 | | | 28.0 | | | | 4.5 | | | | 3.1 | | | | | | 1.3 | | | | | | | | | | 2.2 | | | | | |  |
|  | | | | | | | | %w | 59.7 | | | 27.4 | | | | 5.4 | | | | 3.7 | | | | | | 1.5 | | | | | | | | | | 2.4 | | | | | |  |
| Having physical contact with someone  who has coronavirus but no symptoms | | | | | | | | n | 1932 | | | 305 | | | | 69 | | | | 21 | | | | | | 23 | | | | | | | | | | 43 | | | | | |  |
|  | | | | | | | | % | 80.7 | | | 12.7 | | | | 2.9 | | | | 0.9 | | | | | | 1.0 | | | | | | | | | | 1.8 | | | | | |  |
|  | | | | | | | | %w | 77.9 | | | 14.3 | | | | 3.2 | | | | 1.2 | | | | | | 1.2 | | | | | | | | | | 2.2 | | | | | |  |
| Having physical contact with someone  with coronavirus who has symptoms | | | | | | | | n | 2076 | | | 193 | | | | 54 | | | | 15 | | | | | | 19 | | | | | | | | | | 36 | | | | | |  |
|  | | | | | | | | % | 86.8 | | | 8.1 | | | | 2.3 | | | | 0.6 | | | | | | 0.8 | | | | | | | | | | 1.5 | | | | | |  |
|  | | | | | | | | %w | 83.7 | | | 9.2 | | | | 3.4 | | | | 0.9 | | | | | | 1.1 | | | | | | | | | | 1.8 | | | | | |  |
| Being in close contact (i.e. within 2 meters) to someone who has coronavirus, when they cough or sneeze | | | | | | | | n | 1095 | | | 738 | | | | 253 | | | | 181 | | | | | | 68 | | | | | | | | | | 58 | | | | | |  |
|  | | | | | | | | % | 45.8 | | | 30.8 | | | | 10.6 | | | | 7.6 | | | | | | 2.8 | | | | | | | | | | 2.4 | | | | | |  |
|  | | | | | | | | %w | 43.3 | | | 32.6 | | | | 10.3 | | | | 7.7 | | | | | | 3.4 | | | | | | | | | | 2.7 | | | | | |  |
| Being further away (i.e. further than 2  meters away) to someone who has coronavirus, when they cough or sneeze | | | | | | | | n | 330 | | | 795 | | | | 420 | | | | 520 | | | | | | 249 | | | | | | | | | | 79 | | | | | |  |
|  | | | | | | | | % | 13.8 | | | 33.2 | | | | 17.6 | | | | 21.7 | | | | | | 10.4 | | | | | | | | | | 3.3 | | | | | |  |
|  | | | | | | | | %w | 14.2 | | | 33.5 | | | | 17.6 | | | | 21.0 | | | | | | 10.1 | | | | | | | | | | 3.7 | | | | | |  |
| Contact with contaminated environments  (e.g. surfaces such as lifts, ATMs, etc.) | | | | | | | | n | 1175 | | | 849 | | | | 220 | | | | 71 | | | | | | 35 | | | | | | | | | | 43 | | | | | |  |
|  | | | | | | | | % | 49.1 | | | 35.5 | | | | 9.2 | | | | 3.0 | | | | | | 1.5 | | | | | | | | | | 1.8 | | | | | |  |
|  | | | | | | | | %w | 46.6 | | | 36.2 | | | | 10.0 | | | | 3.6 | | | | | | 1.7 | | | | | | | | | | 1.9 | | | | | |  |
| Consumption of wild animal meat  (e.g. rabbit, venison, pheasant, etc.) | | | | | | | | n | 127 | | | 225 | | | | 499 | | | | 435 | | | | | | 728 | | | | | | | | | | 379 | | | | | |  |
|  | | | | | | | | % | 5.3 | | | 9.4 | | | | 20.9 | | | | 18.2 | | | | | | 30.4 | | | | | | | | | | 15.8 | | | | | |  |
|  | | | | | | | | %w | 5.6 | | | 9.9 | | | | 20.3 | | | | 17.6 | | | | | | 30.5 | | | | | | | | | | 16.0 | | | | | |  |
| Visiting public markets that sell fresh meat, fish or poultry | | | | | | | | n | 311 | | | 715 | | | | 629 | | | | 300 | | | | | | 236 | | | | | | | | | | 202 | | | | | |  |
|  | | | | | | | | % | 13.0 | | | 29.9 | | | | 26.3 | | | | 12.5 | | | | | | 9.9 | | | | | | | | | | 8.4 | | | | | |  |
|  | | | | | | | | %w | 13.0 | | | 30.8 | | | | 25.8 | | | | 11.8 | | | | | | 10.8 | | | | | | | | | | 7.8 | | | | | |  |
| Consumption of seafood imported from  China | | | | | | | | n | 334 | | | 397 | | | | 550 | | | | 308 | | | | | | 458 | | | | | | | | | | 346 | | | | | |  |
|  | | | | | | | | % | 14.0 | | | 16.6 | | | | 23.0 | | | | 12.9 | | | | | | 19.1 | | | | | | | | | | 14.5 | | | | | |  |
|  | | | | | | | | %w | 14.1 | | | 17.4 | | | | 22.2 | | | | 12.1 | | | | | | 20.8 | | | | | | | | | | 13.3 | | | | | |  |
| Consumption/ use of products imported  from China | | | | | | | | n | 262 | | | 369 | | | | 571 | | | | 385 | | | | | | 519 | | | | | | | | | | 287 | | | | | |  |
|  | | | | | | | | % | 10.9 | | | 15.4 | | | | 23.9 | | | | 16.1 | | | | | | 21.7 | | | | | | | | | | 12.0 | | | | | |  |
|  | | | | | | | | %w | 12.2 | | | 16.3 | | | | 22.6 | | | | 14.6 | | | | | | 22.6 | | | | | | | | | | 11.7 | | | | | |  |
| **Which, if any, of the following measures have you personally taken to protect yourself and/or others from the coronavirus (i.e. COVID-19)? (Please select all that apply)** | | | | | | | | | | | | | | | | | | | | | | | | | | | | | | | | | | | | | | | | | |  |
|  | | | | | | | | | | | | | | *Measures taken to protect* | | | | | | | | | | | | | | | | | | | | | | | | | | | |  |
|  | | | | | | | | | | | | | | ***myself*** | | | | | | | | | | | | | ***others*** | | | | | | | | | | | | | | |  |
|  | | | | | | | | | | | | | | ***n*** | | | | ***%*** | | | | ***%w*** | | | | | ***n*** | | | | ***%*** | | | | ***%w*** | | | | | | |  |
| Worn a face mask - | | | | | | | | | | | | | | 1955 | | | | 81.7 | | | | 83.0 | | | | | 1348 | | | | 56.3 | | | | 55.9 | | | | | | |  |
| Washed hands more frequently with soap and water | | | | | | | | | | | | | | 2316 | | | | 96.8 | | | | 95.6 | | | | | 1187 | | | | 49.6 | | | | 50.7 | | | | | | |  |
| Used hand sanitiser more regularly | | | | | | | | | | | | | | 1964 | | | | 82.1 | | | | 82.8 | | | | | 985 | | | | 41.2 | | | | 42.3 | | | | | | |  |
| Disinfected my home | | | | | | | | | | | | | | 1766 | | | | 73.8 | | | | 73.1 | | | | | 1126 | | | | 47.1 | | | | 45.5 | | | | | | |  |
| Covered my nose and mouth when sneezing or coughing | | | | | | | | | | | | | | 1708 | | | | 71.4 | | | | 72.1 | | | | | 1592 | | | | 66.5 | | | | 65.6 | | | | | | |  |
| Avoided contact with people who have a fever or respiratory symptoms (e.g. a cough) | | | | | | | | | | | | | | 2049 | | | | 85.6 | | | | 87.3 | | | | | 977 | | | | 40.8 | | | | 41.8 | | | | | | |  |
| Avoided contact with people who have been to affected areas within the last 14 days | | | | | | | | | | | | | | 1949 | | | | 81.4 | | | | 82.4 | | | | | 911 | | | | 38.1 | | | | 38.8 | | | | | | |  |
| Avoided going out in general | | | | | | | | | | | | | | 2148 | | | | 89.8 | | | | 87.1 | | | | | 1212 | | | | 50.6 | | | | 48.9 | | | | | | |  |
| Avoided crowded areas | | | | | | | | | | | | | | 2223 | | | | 92.9 | | | | 92.3 | | | | | 1201 | | | | 50.2 | | | | 48.8 | | | | | | |  |
| Avoided going to public markets that sell fresh meat. fish or poultry | | | | | | | | | | | | | | 1928 | | | | 80.6 | | | | 78.2 | | | | | 947 | | | | 39.6 | | | | 39.1 | | | | | | |  |
| Avoided going to hospital or other healthcare settings | | | | | | | | | | | | | | 2000 | | | | 83.6 | | | | 81.0 | | | | | 986 | | | | 41.2 | | | | 41.1 | | | | | | |  |
| Avoided taking public transport | | | | | | | | | | | | | | 2011 | | | | 84.0 | | | | 83.6 | | | | | 987 | | | | 41.2 | | | | 40.6 | | | | | | |  |
| Avoided going to work | | | | | | | | | | | | | | 1770 | | | | 74.0 | | | | 71.4 | | | | | 926 | | | | 38.7 | | | | 39.5 | | | | | | |  |
| Avoided going into shops and supermarkets | | | | | | | | | | | | | | 1685 | | | | 70.4 | | | | 67.1 | | | | | 862 | | | | 36.0 | | | | 35.4 | | | | | | |  |
| Avoided social events | | | | | | | | | | | | | | 2166 | | | | 90.5 | | | | 89.6 | | | | | 1281 | | | | 53.5 | | | | 52.4 | | | | | | |  |
| Avoided travel to affected areas in the world | | | | | | | | | | | | | | 2064 | | | | 86.3 | | | | 85.9 | | | | | 1020 | | | | 42.6 | | | | 42.4 | | | | | | |  |
| Avoided travel to other areas (outside KSA), regardless of whether they're affected | | | | | | | | | | | | | | 2033 | | | | 85.0 | | | | 84.7 | | | | | 1006 | | | | 42.0 | | | | 41.6 | | | | | | |  |
| Avoided travel to other areas (inside KSA), regardless of whether they're affected | | | | | | | | | | | | | | 1967 | | | | 82.2 | | | | 81.9 | | | | | 1003 | | | | 41.9 | | | | 41.7 | | | | | | |  |
| Moved temporarily to the countryside or a remote location | | | | | | | | | | | | | | 1045 | | | | 43.7 | | | | 43.8 | | | | | 516 | | | | 21.6 | | | | 23.0 | | | | | | |  |
| Don't know/ can't recall | | | | | | | | | | | | | | 219 | | | | 9.2 | | | | 9.0 | | | | | 108 | | | | 4.5 | | | | 6.3 | | | | | | |  |
| Not applicable | | | | | | | | | | | | | | 233 | | | | 9.7 | | | | 9.6 | | | | | 113 | | | | 4.7 | | | | 6.2 | | | | | | |  |
|  | | | | | | | | | | | | | | | | | | | | | | | | **n** | | | | | | **%** | | | | | **%w** | | | |  |  |  |  |
| **Other measures taken to protect yourself and/or others, please specify** | | | | | | | | | | | | | | | | | | | | | | | |  | | | | | |  | | | | |  | | | |  |  |  |  |
|  | | | | | | | | | | | | | | | | | | | | | | | | 261 | | | | | | 10.9 | | | | | 11.0 | | | |  |  |  |  |
| **You previously mentioned that you have changed your behaviour to protect yourself and/or others from coronavirus (i.e. COVID-19)...Which, if any, of the following was this in response to? (Please select all that apply.** | | | | | | | | | | | | | | | | | | | | | | | | | | | | | | | | | | | | | | | | | |  |
|  | | | | | | | | | | | | | | | | | | | | **n** | | | | | | | | **%** | | | | | | **%w** | | | | | |  |  |  |
| In response to the KSA government guidance | | | | | | | | | | | | | | | | | | | | 2202 | | | | | | | | 92.0 | | | | | | 92.9 | | | | | |  |  |  |
| In response to news coverage of the outbreak | | | | | | | | | | | | | | | | | | | | 1212 | | | | | | | | 50.6 | | | | | | 51.5 | | | | | |  |  |  |
| In response to the first death to COVID-19 in KSA | | | | | | | | | | | | | | | | | | | | 497 | | | | | | | | 20.8 | | | | | | 19.1 | | | | | |  |  |  |
| In response to the first death to COVID-19 in Europe | | | | | | | | | | | | | | | | | | | | 142 | | | | | | | | 5.9 | | | | | | 6.0 | | | | | |  |  |  |
| In response to the first death to COVID-19 in China | | | | | | | | | | | | | | | | | | | | 134 | | | | | | | | 5.6 | | | | | | 5.3 | | | | | |  |  |  |
| In response to news of people stockpiling | | | | | | | | | | | | | | | | | | | | 199 | | | | | | | | 8.3 | | | | | | 7.8 | | | | | |  |  |  |
| In response to the growing number of COVID-19 cases in KSA | | | | | | | | | | | | | | | | | | | | 1213 | | | | | | | | 50.7 | | | | | | 46.3 | | | | | |  |  |  |
| In response to what is happening in other countries (e.g. Italy, Iran, etc.) | | | | | | | | | | | | | | | | | | | | 953 | | | | | | | | 39.8 | | | | | | 38.2 | | | | | |  |  |  |
| Due to my own personal experience of the outbreak | | | | | | | | | | | | | | | | | | | | 74 | | | | | | | | 3.1 | | | | | | 3.3 | | | | | |  |  |  |
| Don't know/ can't recall | | | | | | | | | | | | | | | | | | | | 44 | | | | | | | | 1.8 | | | | | | 1.5 | | | | | |  |  |  |
| Not applicable- I have not taken any measures to protect myself/others | | | | | | | | | | | | | | | | | | | | 29 | | | | | | | | 1.2 | | | | | | 1.2 | | | | | |  |  |  |
| Other | | | | | | | | | | | | | | | | | | | | 54 | | | | | | | | 2.3 | | | | | | 2.2 | | | | | |  |  |  |
| **In general, how effective or ineffective do you think each of the following measures are in preventing the spread of coronavirus (COVID-19)? (Please select one option on each row)** | | | | | | | | | | | | | | | | | | | | | | | | | | | | | | | | | | | | | | | | | |  |
|  | | | |  | | | *Very effective* | | | | | | *Fairly effective* | | | | *Fairly ineffective* | | | | | | *Very ineffective* | | | | | | | | |  | *Don't know* | | | | | | | |  |  |
|  | | | |  | | |  | | | | | |  | | | |  | | | | | |  | | | | | | | | |  | | | | | |  |  |  |  |  |
| Wearing face masks | | | | n | | | 1334 | | | | | | 890 | | | | 103 | | | | | | 32 | | | | | | | | | 34 | | | | | |  |  |  |  |  |
|  | | | | % | | | 55.7 | | | | | | 37.2 | | | | 4.3 | | | | | | 1.3 | | | | | | | | | 1.4 | | | | | |  |  |  |  |  |
|  | | | | %w | | | 55.5 | | | | | | 36.7 | | | | 4.5 | | | | | | 1.4 | | | | | | | | | 1.8 | | | | | |  |  |  |  |  |
| Washing hands frequently with soap and | | | | n | | | 2019 | | | | | | 322 | | | | 26 | | | | | | 6 | | | | | | | | | 20 | | | | | |  |  |  |  |  |
| water | | | | % | | | 84.4 | | | | | | 13.5 | | | | 1.1 | | | | | | 0.3 | | | | | | | | | 0.8 | | | | | |  |  |  |  |  |
|  | | | | %w | | | 84.3 | | | | | | 13.4 | | | | 0.9 | | | | | | 0.4 | | | | | | | | | 1.1 | | | | | |  |  |  |  |  |
| Using hand sanitiser | | | | n | | | 1582 | | | | | | 686 | | | | 79 | | | | | | 18 | | | | | | | | | 28 | | | | | |  |  |  |  |  |
|  | | | | % | | | 66.1 | | | | | | 28.7 | | | | 3.3 | | | | | | 0.8 | | | | | | | | | 1.2 | | | | | |  |  |  |  |  |
|  | | | | %w | | | 65.7 | | | | | | 28.7 | | | | 3.2 | | | | | | 0.8 | | | | | | | | | 1.6 | | | | | |  |  |  |  |  |
| Disinfecting homes | | | | n | | | 1304 | | | | | | 794 | | | | 197 | | | | | | 42 | | | | | | | | | 56 | | | | | |  |  |  |  |  |
|  | | | | % | | | 54.5 | | | | | | 33.2 | | | | 8.2 | | | | | | 1.8 | | | | | | | | | 2.3 | | | | | |  |  |  |  |  |
|  | | | | %w | | | 52.7 | | | | | | 33.4 | | | | 8.8 | | | | | | 2.0 | | | | | | | | | 3.2 | | | | | |  |  |  |  |  |
| Covering your nose and mouth when | | | | n | | | 1930 | | | | | | 388 | | | | 43 | | | | | | 14 | | | | | | | | | 18 | | | | | |  |  |  |  |  |
| sneezing or coughing | | | | % | | | 80.7 | | | | | | 16.2 | | | | 1.8 | | | | | | 0.6 | | | | | | | | | 0.8 | | | | | |  |  |  |  |  |
|  | | | | %w | | | 80.9 | | | | | | 15.5 | | | | 1.9 | | | | | | 0.8 | | | | | | | | | 0.9 | | | | | |  |  |  |  |  |
| Avoiding contact with people who have a | | | | n | | | 2084 | | | | | | 242 | | | | 34 | | | | | | 7 | | | | | | | | | 26 | | | | | |  |  |  |  |  |
| fever or respiratory symptoms | | | | % | | | 87.1 | | | | | | 10.1 | | | | 1.4 | | | | | | 0.3 | | | | | | | | | 1.1 | | | | | |  |  |  |  |  |
| (e.g. a cough) | | | | %w | | | 86.6 | | | | | | 10.1 | | | | 1.4 | | | | | | 0.4 | | | | | | | | | 1.5 | | | | | |  |  |  |  |  |
| Avoiding contact with people who have | | | | n | | | 2057 | | | | | | 257 | | | | 44 | | | | | | 9 | | | | | | | | | 26 | | | | | |  |  |  |  |  |
| been to affected areas within the last 14 | | | | % | | | 86.0 | | | | | | 10.7 | | | | 1.8 | | | | | | 0.4 | | | | | | | | | 1.1 | | | | | |  |  |  |  |  |
| days | | | | %w | | | 85.5 | | | | | | 11.0 | | | | 1.7 | | | | | | 0.4 | | | | | | | | | 1.4 | | | | | |  |  |  |  |  |
| Avoiding going out in general | | | | n | | | 1705 | | | | | | 513 | | | | 120 | | | | | | 29 | | | | | | | | | 26 | | | | | |  |  |  |  |  |
|  | | | | % | | | 71.2 | | | | | | 21.4 | | | | 5.0 | | | | | | 1.2 | | | | | | | | | 1.1 | | | | | |  |  |  |  |  |
|  | | | | %w | | | 68.8 | | | | | | 22.6 | | | | 5.8 | | | | | | 1.4 | | | | | | | | | 1.4 | | | | | |  |  |  |  |  |
| Avoiding crowded areas | | | | n | | | 2049 | | | | | | 274 | | | | 43 | | | | | | 10 | | | | | | | | | 17 | | | | | |  |  |  |  |  |
|  | | | | % | | | 85.6 | | | | | | 11.5 | | | | 1.8 | | | | | | 0.4 | | | | | | | | | 0.7 | | | | | |  |  |  |  |  |
|  | | | | %w | | | 83.7 | | | | | | 12.9 | | | | 2.1 | | | | | | 0.3 | | | | | | | | | 1.0 | | | | | |  |  |  |  |  |
| Avoiding going to public markets that sell | | | | n | | | 1431 | | | | | | 550 | | | | 224 | | | | | | 59 | | | | | | | | | 129 | | | | | |  |  |  |  |  |
| fresh meat, fish or poultry | | | | % | | | 59.8 | | | | | | 23.0 | | | | 9.4 | | | | | | 2.5 | | | | | | | | | 5.4 | | | | | |  |  |  |  |  |
|  | | | | %w | | | 59.3 | | | | | | 23.7 | | | | 9.8 | | | | | | 2.2 | | | | | | | | | 5.0 | | | | | |  |  |  |  |  |
| Avoiding going to hospital or other | | | | n | | | 1605 | | | | | | 555 | | | | 152 | | | | | | 29 | | | | | | | | | 52 | | | | | |  |  |  |  |  |
| healthcare settings | | | | % | | | 67.1 | | | | | | 23.2 | | | | 6.4 | | | | | | 1.2 | | | | | | | | | 2.2 | | | | | |  |  |  |  |  |
|  | | | | %w | | | 64.7 | | | | | | 24.6 | | | | 6.9 | | | | | | 1.3 | | | | | | | | | 2.5 | | | | | |  |  |  |  |  |
| Avoiding taking public transport | | | | n | | | 1869 | | | | | | 387 | | | | 77 | | | | | | 13 | | | | | | | | | 47 | | | | | |  |  |  |  |  |
|  | | | | % | | | 78.1 | | | | | | 16.2 | | | | 3.2 | | | | | | 0.5 | | | | | | | | | 2.0 | | | | | |  |  |  |  |  |
|  | | | | %w | | | 76.7 | | | | | | 16.6 | | | | 3.7 | | | | | | 0.6 | | | | | | | | | 2.5 | | | | | |  |  |  |  |  |
| Avoiding going to work | | | | n | | | 1413 | | | | | | 573 | | | | 239 | | | | | | 70 | | | | | | | | | 98 | | | | | |  |  |  |  |  |
|  | | | | % | | | 59.0 | | | | | | 23.9 | | | | 10.0 | | | | | | 2.9 | | | | | | | | | 4.1 | | | | | |  |  |  |  |  |
|  | | | | %w | | | 55.7 | | | | | | 25.7 | | | | 10.6 | | | | | | 3.5 | | | | | | | | | 4.5 | | | | | |  |  |  |  |  |
| Avoiding going to school or avoid letting | | | | n | | | 1807 | | | | | | 402 | | | | 103 | | | | | | 26 | | | | | | | | | 55 | | | | | |  |  |  |  |  |
| your children go to school | | | | % | | | 75.5 | | | | | | 16.8 | | | | 4.3 | | | | | | 1.1 | | | | | | | | | 2.3 | | | | | |  |  |  |  |  |
|  | | | | %w | | | 73.0 | | | | | | 18.3 | | | | 4.6 | | | | | | 1.2 | | | | | | | | | 2.8 | | | | | |  |  |  |  |  |
| Avoiding going into shops and supermarkets | | | | n | | | 1405 | | | | | | 700 | | | | 201 | | | | | | 46 | | | | | | | | | 41 | | | | | |  |  |  |  |  |
|  | | | | % | | | 58.7 | | | | | | 29.3 | | | | 8.4 | | | | | | 1.9 | | | | | | | | | 1.7 | | | | | |  |  |  |  |  |
|  | | | | %w | | | 56.0 | | | | | | 31.1 | | | | 8.9 | | | | | | 2.0 | | | | | | | | | 2.0 | | | | | |  |  |  |  |  |
| Avoiding social events | | | | n | | | 2035 | | | | | | 274 | | | | 47 | | | | | | 12 | | | | | | | | | 25 | | | | | |  |  |  |  |  |
|  | | | | % | | | 85.0 | | | | | | 11.5 | | | | 2.0 | | | | | | 0.5 | | | | | | | | | 1.0 | | | | | |  |  |  |  |  |
|  | | | | %w | | | 83.8 | | | | | | 12.0 | | | | 2.3 | | | | | | 0.6 | | | | | | | | | 1.4 | | | | | |  |  |  |  |  |
| Avoiding travel to affected areas in the | | | | n | | | 2117 | | | | | | 210 | | | | 35 | | | | | | 9 | | | | | | | | | 22 | | | | | |  |  |  |  |  |
| world | | | | % | | | 88.5 | | | | | | 8.8 | | | | 1.5 | | | | | | 0.4 | | | | | | | | | 0.9 | | | | | |  |  |  |  |  |
|  | | | | %w | | | 87.1 | | | | | | 9.1 | | | | 2.0 | | | | | | 0.5 | | | | | | | | | 1.3 | | | | | |  |  |  |  |  |
| Avoiding travel to other areas (outside | | | | n | | | 1965 | | | | | | 306 | | | | 69 | | | | | | 17 | | | | | | | | | 36 | | | | | |  |  |  |  |  |
| KSA), regardless | | | | % | | | 82.1 | | | | | | 12.8 | | | | 2.9 | | | | | | 0.7 | | | | | | | | | 1.5 | | | | | |  |  |  |  |  |
|  | | | | %w | | | 79.4 | | | | | | 14.0 | | | | 3.7 | | | | | | 1.0 | | | | | | | | | 1.8 | | | | | |  |  |  |  |  |
| Avoiding travel to other areas (inside | | | | n | | | 1859 | | | | | | 369 | | | | 100 | | | | | | 23 | | | | | | | | | 42 | | | | | |  |  |  |  |  |
| KSA), regardless of whether they're | | | | % | | | 77.7 | | | | | | 15.4 | | | | 4.2 | | | | | | 1.0 | | | | | | | | | 1.8 | | | | | |  |  |  |  |  |
| affected | | | | %w | | | 73.7 | | | | | | 18.3 | | | | 4.8 | | | | | | 1.1 | | | | | | | | | 2.2 | | | | | |  |  |  |  |  |
| Moving temporarily to the countryside | | | | n | | | 981 | | | | | | 505 | | | | 424 | | | | | | 194 | | | | | | | | | 289 | | | | | |  |  |  |  |  |
| or a remote location | | | | % | | | 41.0 | | | | | | 21.1 | | | | 17.7 | | | | | | 8.1 | | | | | | | | | 12.1 | | | | | |  |  |  |  |  |
|  | | | | %w | | | 42.0 | | | | | | 22.5 | | | | 17.0 | | | | | | 6.8 | | | | | | | | | 11.7 | | | | | |  |  |  |  |  |
| **According to the Ministry of Health, to 'self-isolate' means if you just returned from traveling abroad from certain countries or came in contact with an infected person, do not leave your home (even to buy food or essentials) or have any visitors for 14 days. This includes not going to work, or other public places, and avoiding public transport or taxis. Self-isolation is the same as voluntary quarantine. If you were advised to do so by a healthcare professional, would you be able and willing to self-isolate? (Please select one option on each row).** | | | | | | | | | | | | | | | | | | | | | | | | | | | | | | | | | | |  |  |  |  |  |  |  |  |
|  | | **Able to self-isolate** | | | | | | | | | | | | | | **Willing to self-isolate** | | | | | | | | | | | | | | | | | | |  |  |  |  |  |  |  |  |
|  | | ***n*** | | | ***%*** | | | | | | ***%w*** | | | | | ***n*** | | | ***%*** | | | | | | | | | | ***%w*** | | | | | |  |  |  |  |  |  |  |  |
| Yes, I would | | 2113 | | | 88.3 | | | | | | 87.5 | | | | | 1947 | | | 81.4 | | | | | | | | | | 81.6 | | | | | |  |  |  |  |  |  |  |  |
| No, I wouldn't | | 85 | | | 3.6 | | | | | | 3.5 | | | | | 193 | | | 8.1 | | | | | | | | | | 8.1 | | | | | |  |  |  |  |  |  |  |  |
| Don't know | | 195 | | | 8.1 | | | | | | 9.0 | | | | | 253 | | | 10.6 | | | | | | | | | | 10.3 | | | | | |  |  |  |  |  |  |  |  |
| Total | | 2393 | | | 100.0 | | | | | | 100.0 | | | | | 2393 | | | 100.0 | | | | | | | | | | 100.0 | | | | | |  |  |  |  |  |  |  |  |

| **Which, if any, of the following** **have you done in order to prepare for a potential self-isolation? (Please select all that apply.** | | | | | | |
| --- | --- | --- | --- | --- | --- | --- |
|  | ***n*** | | | ***%*** | | ***%w*** |
| Stocking up on food supplies | 1160 | | | 48.5 | | 49.4 |
| Stocking up on toiletries (e.g. toilet paper, shampoo, soap, hand sanitizer, etc.) | 1000 | | | 41.8 | | 40.6 |
| Stocking up on prescription medicines | 573 | | | 23.9 | | 24.5 |
| Stocking up on over-the-counter medicines (e.g. paracetamol, ibuprofen, etc.) | 574 | | | 24.0 | | 24.5 |
| Establishing remote working capabilities | 817 | | | 34.1 | | 35.5 |
| Finding alternative childcare | 97 | | | 4.1 | | 4.2 |
| Don't know/ can't recall | 140 | | | 5.9 | | 5.4 |
| Not applicable | 889 | | | 37.2 | | 36.8 |
| Other | 70 | | | 2.9 | | 3.2 |
| **Which, if any, of the following** **do you see as potential problems as you are self-isolating? (Please select all that apply).** | |  |  | |  |  |
|  | | | | ***n*** | ***%*** | ***%w*** |
| It would negatively affect my mental health  (e.g. I would get bored, stressed, lonely, anxious, angry, etc.) | | | | 1005 | 42.0 | 39.3 |
| I would find it difficult to separate myself from others in my household  (e.g. my children, housemates, etc.) | | | | 778 | 32.5 | 30.0 |
| I would find it difficult to get supplies, (e.g. food, medicines, etc.) | | | | 506 | 21.1 | 20.8 |
| I have caring responsibilities and would find it difficult to find someone to cover those  (e.g. I care for a dependent child, someone with disabilities, an elderly relative, neighbour, etc.) | | | | 313 | 13.1 | 13.0 |
| It would negatively affect my social life (e.g. it would negatively impact my friendships,  I would miss attending social and cultural events, etc.) | | | | 266 | 11.1 | 10.4 |
| I would experience a loss of income | | | | 185 | 7.7 | 8.8 |
| My studies or education would suffer | | | | 248 | 10.4 | 8.1 |
| Don't know | | | | 177 | 7.4 | 7.8 |
| Not applicable | | | | 792 | 33.1 | 35.7 |
| Other | | | | 31 | 1.3 | 1.2 |

| **How likely or unlikely would you be to NOT report or seek help for symptoms of coronavirus to avoid self-isolation measures in each of the following situations? (Please select one option on each row)** | | | | | | | | | |
| --- | --- | --- | --- | --- | --- | --- | --- | --- | --- |
|  | *If I had mild*  *symptoms*  *(e.g. can go about daily*  *tasks normally)* | | | *If I had moderate*  *symptoms*  *(e.g. may need self-care*  *and rest in bed)* | | | *If I had severe*  *symptoms*  *(e.g. may need care and*  *treatment in hospital)* | | |
|  | ***n*** | ***%*** | ***%w*** | ***n*** | ***%*** | ***%w*** | ***n*** | ***%*** | ***%w*** |
| Very likely | 254 | 10.6 | 10.9 | 146 | 6.1 | 6.4 | 124 | 5.2 | 5.9 |
| Fairly likely | 347 | 14.5 | 14.2 | 220 | 9.2 | 10.4 | 51 | 2.1 | 3.1 |
| Fairly unlikely | 334 | 14.0 | 13.7 | 376 | 15.7 | 15.8 | 81 | 3.4 | 3.5 |
| Very unlikely | 1342 | 56.1 | 56.2 | 1568 | 65.5 | 63.4 | 2044 | 85.4 | 83.2 |
| Don't know | 116 | 4.8 | 5.1 | 83 | 3.5 | 3.9 | 93 | 3.9 | 4.3 |

| **Which, if any, of the following** **have you personally experienced/ witnessed in relation to coronavirus (i.e. COVID-19)? (Please select all that apply)** | | | |
| --- | --- | --- | --- |
|  | ***n*** | ***%*** | ***%w*** |
| Harassment / discrimination | 259 | 10.8 | 9.9 |
| Spread of misinformation about the disease or outbreak | 1381 | 57.7 | 53.5 |
| Racist actions | 431 | 18.0 | 15.7 |
| Discrimination against a group based on background or country of origin | 610 | 25.5 | 21.3 |
| Hostility towards someone taking preventative measures | 409 | 17.1 | 13.7 |
| Violence | 97 | 4.1 | 3.6 |
| Other anti-social behaviours | 237 | 9.9 | 8.8 |
| None of these | 520 | 21.7 | 25.2 |
| Don't know/ can't recall | 341 | 14.2 | 14.6 |
| Prefer not to say | 50 | 2.1 | 2.3 |
